# Remote ischemic conditioning: a promising therapeutic intervention for multi-organ protection

**DOI:** 10.18632/aging.101527

**Published:** 2018-08-16

**Authors:** Da Zhou, Jiayue Ding, Jingyuan Ya, Liqun Pan, Yuan Wang, Xunming Ji, Ran Meng

**Affiliations:** 1 Department of Neurology, Xuanwu Hospital, Capital Medical University, Beijing, China; 2 Department of Neurosurgery, Xuanwu Hospital, Capital Medical University, Beijing, China; 3 Center of Stroke, Beijing Institute for Brain Disorders, Beijing, China; 4 Department of China-America Institute of Neuroscience, Xuanwu Hospital, Capital Medical University, Beijing, China; 5 National Clinical Research Center for Geriatric Disorders, Beijing, China; * Equal contribution

**Keywords:** ischemia-reperfusion injury, age-related arteriosclerotic vascular disease, remote ischemic conditioning, clinical translation, multi-organ protection

## Abstract

Despite decades of formidable exploration, multi-organ ischemia-reperfusion injury (IRI) encountered, particularly amongst elderly patients with clinical scenarios, such as age-related arteriosclerotic vascular disease, heart surgery and organ transplantation, is still an unsettled conundrum that besets clinicians. Remote ischemic conditioning (RIC), delivered via transient, repetitive noninvasive IR interventions to distant organs or tissues, is regarded as an innovative approach against IRI. Based on the available evidence, RIC holds the potential of affording protection to multiple organs or tissues, which include not only the heart and brain, but also others that are likely susceptible to IRI, such as the kidney, lung, liver and skin. Neuronal and humoral signaling pathways appear to play requisite roles in the mechanisms of RIC-related beneficial effects, and these pathways also display inseparable interactions with each other. So far, several hurdles lying ahead of clinical translation that remain to be settled, such as establishment of biomarkers, modification of RIC regimen, and deep understanding of underlying minutiae through which RIC exerts its powerful function. As this approach has garnered an increasing interest, herein, we aim to encapsulate an overview of the basic concept and postulated protective mechanisms of RIC, highlight the main findings from proof-of-concept clinical studies in various clinical scenarios, and also to discuss potential obstacles that remain to be conquered. More well designed and comprehensive experimental work or clinical trials are warranted in future research to confirm whether RIC could be utilized as a non-invasive, inexpensive and efficient adjunct therapeutic intervention method for multi-organ protection.

## Introduction

Early restoration of blood flow has been demonstrated to bring substantial benefits via salvaging viable tissues from ischemic injury [1]. However, rapid reperfusion after a certain period of ischemia could paradoxically induce detrimental effects including ischemia-reperfusion injury (IRI), which is known as a major cause of organ dysfunction following ischemic events [2]. IRI is quite common in clinical settings such as thrombolysis after ischemic stroke or myocardial infarction secondary to arteriosclerotic vascular diseases, cardiac surgery and organ transplantation. However, the clinical outcome of the existing approaches to control IRI remains unsatisfactory. Moreover, as the average human lifespan has markedly increased, so has the burden of ageing and age-related disorders on the individuals, which often inflict multiple organs of the elderly with decreased physiological reserve and tissue resilience.

Recently, attention has been focused on an innovative approach, termed as ischemic conditioning (IC), particularly remote ischemic conditioning (RIC), knowing that repetitive, transient and sublethal series of IR bursts can trigger endogenous protection and tolerance against subsequent ischemic threats [3]. RIC may benefit multiple organs of the body at the same time. It seems to be a promising non-pharmaceutical and non-surgical therapy for preventing and treating age-related systemic vascular diseases such as combined lesions in the brain, heart and kidney, and also arteriosclerosis-induced neurodegenerative disorders.

**Figure 1:**
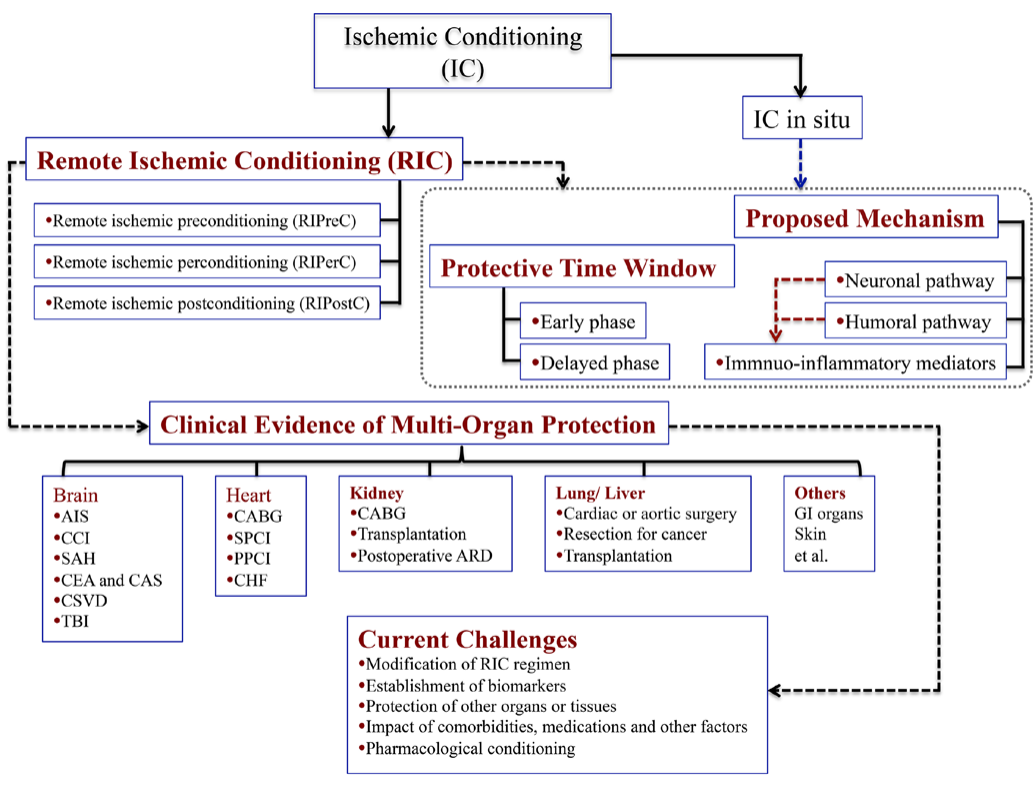
**Schematic diagram showing the main points pertaining to the study.** Abbreviations: AIS, acute ischemic stroke; CCI, Chronic cerebral ischemia; SAH, subarachnoid hemorrhage; CEA, carotid endarterectomy; CAS, carotid angioplasty and stenting; CSVD, cerebral small vessel disease; TBI, traumatic brain injury; CABG, coronary artery bypass graft surgery; SPCI, selective percutaneous coronary intervention; PPCI, primary percutaneous coronary intervention; CHF, chronic heart failure; ARD, acute renal dysfunction.

In this regard, this article encapsulates an overview of IC on multi-organ protection, including a brief developmental history of IC, intervention forms, underlying mechanisms and current clinical evidence as a promising option to attenuate IRI, (Fig. 1). It also discusses some controversies in the latest research in this field.

## RESULTS

### Forms of IC

Based on the sites of intervention, IC strategies can be classified into two forms, that is, in situ and in remote organs or tissues.

### *IC in situ*

IC in situ involves repeated IR intervention applied locally to the organ or tissue itself, thus offering subsequent protection against IRI-induced lethal injury. The concept of IC in situ was first demonstrated by Murry et al. in 1986, who reported that 4 cycles of 5-min occlusion of the circumflex coronary artery interspersed with 5-min reperfusion could significantly reduce the myocardial infarct size induced by a subsequent longer period occlusion of the same vessel [4]. A systemic review of the role of IC in situ in cardiac surgery summarized data from 22 eligible trials with 933 patients, denoting that local IC might be associated with substantial reductions in ventricular arrhythmia, inotrope requirement, and intensive care unit (ICU) stay [5].

Although direct IC has been discovered for decades and holds the potential in ameliorating IRI, its clinical application is still confronted with great challenges: IC in situ requires a direct intervention employed to the target tissue/organ, which is impractical or even invasive in the cases of IRI of internal organs without surgical issues. Therefore, it would be tempting to discover a way through which an equivalent extent of protection might be brought about without invasive intervention on the targeted organs or tissues.

### *RIC*

RIC refers to a brief, repetitive and sublethal IR applied to an organ or tissue to induce global endogenous tolerance and protect distant (remote) target organs or tissues against subsequent prolonged IRI, meaning that the protective effect of IC can be induced not only in situ directly, but also in distant organs [3].

**Figure 2:**
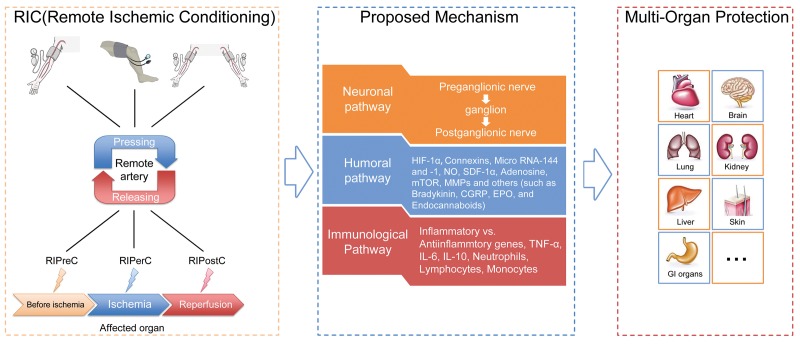
**General illustration of remote ischemic conditioning (RIC).** Remote ischemic conditioning, in which transient sublethal episodes of ischemia and reperfusion are applied to a limb (upper arm or thigh) or limbs, can be delivered before (remote ischemic preconditioning), during (remote ischemic perconditioning) or after (remote ischemic postconditioning) a subsequent and potentially lethal ischemic attack. Neuronal, humoral as well as immunological mediators are postulated to exert critical roles in the transduction of protective signals generated from limbs and surrounding structures to the targeted organs or tissues. The application of RIC has been extended from initially reducing cardiac infarct sizes resulting from acute myocardial infarction to providing protection for a diversity of organs or tissues (other than the heart), which are likely susceptible to ischemia-reperfusion injury, such as the brain, kidney, lung, liver and skin.

As reported by previous studies, there are three methods of RIC intervention based on timing of RIC in relation to IRI, including remote ischemic preconditioning (RIPreC, initiated before IRI), remote ischemic perconditioning (RIPerC, initiated at the moment of ischemia) and remote ischemic postconditioning (RIPostC, initiated at reperfusion stage), (Fig. 2) [3].

*RIPreC.* A landmark study for the breakthrough of IC in situ in the possible clinical application was conducted by Przyklenk et al. in 1993, who used 4 cycles of 5-min IR of the circumflex coronary artery prior to the left anterior descending coronary artery (LADCA) occlusion to reduce myocardial infarction volume at the territory of LADCA in a dog model [6]. This protection induced by remote intra-organ preconditioning drew forth the notion of RIPreC and inspired further investigations to clarify this protective effect. Remote inter-organ preconditioning protection was later proved by in a rat model in 1996, showing that a short period of preconditioning forced upon the renal and mesenteric artery reduced the myocardial infarction [7]. A further study using rabbits revealed that a combination of stimulating the gastrocnemius muscle and restricting femoral blood flow could significantly attenuate IRI-induced cardiac injury, indicating the possibility of inducing preconditioning stimulation by using clinically more applicable method such as the limb tourniquet or other occlusion devices [8]. In 2002, Kharbanda et al. carried out two experiments and found that: 3 bouts of 5-min ischemia/reperfusion induced by human upper limb RIPreC could protect the contralateral forearm from endothelial IRI, and 4 bouts of 5-min lower limb RIPreC could elicit a reduction in the extent of myocardial injury against previously sustained infarction in a swine model [9]. These findings facilitated the translation of RIPreC from experimental models to clinical studies. The first successful pilot human study was conducted by Cheung et al. in 2006 in pediatric patients who underwent repair of congenital heart failure [10]. The researchers observed less potential myocardial injury in the RIPreC group as compared with the control group. Subsequent experimental or clinical investigations on RIPreC demonstrated valuable potential of ameliorating IRI in multiple organs.

*RIPerC.* Another subtype of RIC is RIPerC, which is referred to as the induction of sublethal conditioning stimuli during an episode of ischemic attacks. Schmidt et al. first demonstrated RIPerC by applying cyclical hindlimb IR with a tourniquet in experimental pigs during LADCA occlusion, with a satisfactory result [11]. In two small clinical trials led by Rentoukas and Li, myocardial insults were attenuated among coronary artery disease patients who received RIPerC [12,13]. One classic single-center RCT published in 2010 demonstrated that RIPerC improved myocardial salvage in suspected ST segment elevated myocardial infarction (STEMI) when it was applied during ambulance transport before hospital admission [14].

*RIPostC.* The history of RIPostC dates back to the first report aiming to investigate the cardioprotection of ischemic postconditioning in 2003, showing that brief repetitive ischemia before fully establishment of flow in a canine model of coronary occlusion-reperfusion could attenuate reperfusion-associated myocardial injury [15]. Two years later in 2005, Staat et al. reported the first clinical trial, that is, postconditioning employed to patients undergoing coronary angioplasty for STEMI might provide protection to hearts [16]. Subsequent publications also demonstrated encouraging outcomes of ischemic postconditioning in patients undergoing cardiac surgeries, as presented by reduced cardiac enzyme levels, morbidity, inotrope scores and the duration of ICU stay [17–19].

Selective meta-analyses published up to December 2014 illustrated the potential salutary effects of postconditioning on cardioprotection in subjects with STEMI scheduled for percutaneous coronary intervention (PCI), although some results might be disappointing or even contradictory [20–22]. Unfortunately, the largest clinical trial so far (DANAMI-3–iPOST) concluded that 4 cycles of IC (30-second balloon occlusion followed by 30-second perfusion) during PCI failed to reduce the primary composite outcome of all-cause death and hospitalization due to heart failure in patients with STEMI [23]. One explanation for this neutral result is the algorithm of IC, which differed from previously reported one (4 cycles of 60-second of occlusion followed by 60-second of reperfusion).

### Protective time window

Murry et al. reported that the positive effect of IC diminished or even run out after a few hours, suggesting the existence of a time window for the protection [4]. Subsequent studies verified the above postulation and put forward the theory of two time windows for protection: the first one acted rapidly, but dissipated within 2-3 h; the second one reoccurred after 12-24 h and persisted for up to 3 days [24–27]. Likewise, protective time windows were also reported in animal studies with ischemic perconditioning, demonstrating that the first period occurred immediately after the procedure and persisted for 3-6 h; the second period reoccurred after 24 h and dissipated within 4 days [28,29]. The early or rapid protective phase is believed to be related to changes in intracellular kinase signaling pathways, especially the post-translational modifications of certain proteins. The delayed phase, however, is more likely the result of de novo protein synthesis from dormant genes involved in stress-response, inflammation, angiogenesis, and vasomotor control [30,31]. Nonetheless, unlike RIPreC and RIPerC, scant research has elucidated the therapeutic windows of RIPostC. An animal study revealed that ischemic postconditioning initiated up to 6 h after reperfusion could still confer protection against focal IRI [32]. Subsequent experimental evidence was roughly in agreement with this observation, revealing that RIPostC conducted at 6 h after reperfusion could robustly reduce cerebral infarct volume and ameliorate neurological deficits [33].

### Proposed mechanism of action

**Figure 3:**
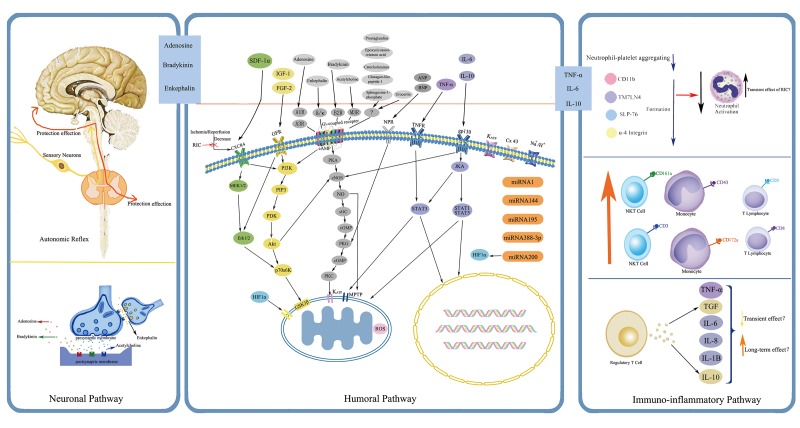
**Simplified overview of the protective pathways of Remote Ischemic Conditioning (RIC).** eNOS/PKG pathway is presented in gray, RISK pathway in yellow and green, and SAFE pathway in purple. Abbreviations: RISK, reperfusion injury salvage kinase; SAFE, survivor activating factor enhancement; SDF-1α, stromal cell derived factor-1α; IGF-1, insulin like growth factor-1; FGF-2: fibroblast growth factor-2; ANP, atrial natriuretic peptide; BNP, brain natriuretic peptide; TNF-α, tumor necrosis factor-α; IL-6, interleukin-6; IL-10, interleukin-10; CXCR4, chemokine 4 receptor; GFR, growth factor receptor; A1R, A3R, adenosine receptor A1, A3; δ/κ, δ- and κ- opioid receptor; B2R, bradykinin receptor B2; M3R, muscarinic receptor M3; NPR, natriuretic peptide receptor; TNFR, tumor necrosis factor receptor; gp130, glycoprotein 130; KATP, ATP-dependent potassium channel; Cx 43, connexin43; MEK1/2, also known as mitogen-activated protein kinase kinase 1/2; Erk1/2, extracellular-regulated kinases 1/2; PI3K, phosphatidylinositol-4, 5-bisphosphate3-kinase; PIP3, phos-phatidylinositol-3, 4, 5-biphosphate; PDK, phosphatidylinositol kinase; Akt, also known as protein kinase B; P70s6K, p70 ribosomal protein s6 kinase; GSK3β, glycogen synthase kinase 3β; HIF-1α, hypoxia inducible factor-1α; cAMP, cyclic adenosine monophosphate; PKA, protein kinase A; eNOS, endothelial nitric oxide synthase; NO, nitric oxide; sGC, soluble guanylate cyclase; cGMP, cyclic guanine monophosphate; PKG, protein kinase G; PKC, protein kinase C; MPTP, mitochondrial permeability transition pore; JKA, Janus kinase; STAT1, STAT3, STAT5, signal transducer and activator of transcription 1, 3, 5; ROS, reactive oxygen species; miRNA, microRNA.

The protective mechanisms of RIC through which the protection is transferred to remote targeted organs against IRI are quite sophisticated and have not been fully explored. Current data reveal that the possible underlying mechanisms are correlated with several aspects, mainly including neuronal and humoral pathways, both of which also display inseparable interactions with each other, and the dominant one may depend on the applied stimuli and specific circumstances [29]. Although most observations have thus far been derived from preclinical animal studies focusing with cardioprotection, they might shed light on the exploration of RIC mechanisms in humans. Here below, a precise overview pertaining to some relatively important pathways and associated mediators are recapitulated, (Fig. 2 and Fig. 3).

### *Neuronal pathway*

The neuronal mechanism has been proposed to play a requisite role in RIC, whereby protective signals from the periphery limbs can be transferred to ischemic organs. A growing body of evidence supports this neuronal hypothetic notion of RIC. For instance, the use of ganglionic blockers such as hexamethonium and trimetaphan could abrogate the RIC-mediated protection in rat models or humans [7,27,34]. Cardioprotection induced by ischemic preconditioning in remote vessels, such as the femoral, renal, and mesenteric arteries has been confirmed in experimental studies, while subsequent transection of the corresponding nerves was able to blunt this effect, suggesting the necessity of afferent nerves for protective signaling transduction [35,36]. In addition, pretreatment with afferent nerve blocker-capsaicin attenuated or even reversed the beneficial effect of RIC in several models, including cerebral, gastric and intestinal ischemic models [37–39]. Moreover, spinal cord reflexes might also exert essential functions in the neuronal pathways, with the supporting evidence that transection of the spinal cord at T9-T10 level and selective blockage of the spinal opioid receptor with naloxone methiodide abolished the RIC effect [35,40]. With regard to the efferent outflow, cardiac studies suggested the crucial role of the parasympathetic nervous system for cardioprotection provided by RIC [41,42]. Although there is still a lack of evidence regarding cerebrovascular diseases, experimental studies in the rat stroke model demonstrated that stimulation of parasympathetic nerve, especially the vagus nerve, could exert a neuroprotective function [43,44]. The function of parasympathetic outflow in RIC was also demonstrated in a human trial by Enko et al. [45].

### *Humoral pathway*

*Humoral mediators.* The humoral pathway hypothesis refers to repeated cycles of IC at a distant site (for example at limbs) may stimulate the release of certain substances that travel into the blood circulation and then reach the prolonged ischemic organs/tissues to produce a protective effect [46]. This hypothesis was firstly highlighted by a study, which found that the cardioprotective effect of ischemic preconditioning could be transferred from preconditioned to non-preconditioned naïve rabbits through whole blood transfusion [47]. In a porcine transplant model, a study group preconditioned the recipient pigs with limb RIC before receiving hearts from brain-dead donors and ultimately observed a 57% reduction in postoperative cardiac infarct lesions, suggesting that some sort of circulating factors after RIC probably mediated the cardioprotection, even in the absence of intact neural innervation (denervated transplanted hearts) [48]. Shimizu et al. later performed several experiments in order to investigate the humoral nature of limb RIC and its effects on isolated rabbit heart models [11]. The results showed that transferring plasma and dialysate (with 15 kDa dialysis membrane) harvested from donor rabbits undergoing limb-RIC conferred protection to non-preconditioned animals; nonetheless, this phenomenon vanished in subjects who were pretreated with naloxone, implying the possible participation of the opioid receptor pathway. Afterwards, experimental studies, specifically those adopting Langendorff bioassays, proposed that this protection was attributed to unidentified hydrophobic, thermolabile circulating proteins with molecular weights ranging from 3.5 to 15-30 kDa [49,50]. Notably, the presence of protective factors after RIC has also been explored in humans, denoting the results as follows: plasma dialysate from diabetic patients without periphery neuropathy who underwent RIC could confer a cardioprotective effect to rabbit hearts suffering IRI, whereas, this effect was suppressed in hearts immersed with RIC dialysate from diabetic patients concurrent with peripheral neuropathy [51]. A recent study reported that total subdiaphragmatic vagotomy, gastric vagotomy, and selective sectioning of the posterior gastric branch abrogated RIC cardioprotection, suggesting the potentially crucial role of the posterior gastric branch of the vagus nerve in the innervation of circulating factors elicited after RIC [52]. Taken together, RIC might promote the release of some sort of or multiple circulating protective factors that are transferrable between species; an intact neural pathway might be necessarily required for RIC-induced protection; and an tight interaction may exist between the neural and humoral pathways, though details need to be further elucidated.

Despite the clinical importance, actual identification of humoral factors following conditioning response faces great challenges and remains ambiguous. Many proteomic analyses using plasma harvested from both animals and humans undergoing RIC have yielded inconsistent or even paradoxical results. A variety of circulating factors have been proposed as contributors, such as hypoxia inducible factor-1α [53–60], connexin 43 [61–64], microRNA [65–75], nitric oxide [76–82], stromal derived factor-1α [83–86], mammalian target of rapamycin [87–89], matrix metalloproteinases [90–94], adenosine [95–104], bradykinin [105–108], erythropoietin [109], endocannabinoids [110], kallikrein [23], and neuroglobin [111], (Fig. 2). Before being put into clinical application, these potential biomarkers should be reassessed and validated with respect to their efficiency, possible mechanism of action, the changing mode of genomics and proteomics following different conditioning regimens, the time interval that can be detected, and compatibility with RIC-mediated protection, all of which are intriguing but full of challenges.

*Immuno-inflammatory responses.* Emerging evidence plunks for the fact that induction of endogenous protection via RIC is partially attributed to the modulation of immuno-inflammatory responses. Konstantinov et al. initially discovered that RIC in healthy humans was capable of downregulating proinflammatory genes while upregulating anti-inflammatory genes, around 30 of which were supposed to be involved in leucocyte adhesion, chemotaxis, cell adherence/migration, apoptosis, TNF-α signaling pathway, and Toll-like receptor pathway [112]. Of particular interest, transient unilateral hindlimb ischemia induced in mice could modify myocardial gene expression in response to oxidative stress, inflammation and mitochondrial function at both early and later phases following the procedure [113]. On the one hand, the expressions of proinflammatory genes such as Egr-1 and Dusp 1/6 were decreased. On the other hand, genes involved in the attenuation of oxidative stress response such as HADHSC and peroxiredoxin-4 were strengthened, and those with the potential of aggravating oxidative injury like PDGFRB and Erp57 were suppressed. Particularly, RIC-mediated protection was reflected by reduced neutrophil activation and leukocyte-endothelium interactions [114,115]. However, Albrecht et al. attributed the cardioprotection of RIC to increased infiltration of neutrophils [59]. Similarly, contradictory results were also reported, saying that TNF-α was increased in some RIC models and reduced in others [59,103,115,116]. It may be difficult to explain these seemingly discrepancies about the inflammatory mediators in RIC studies at present in that either neutrophils or TNF-α has been implicated in providing both protective and detrimental effects [117,118]. Additionally, it may be due to limited time points and different experimental subjects or protocols designed for detecting these mediators. The interactions among diverse signal cascades underlying RIC are still obscure. Although IL-6 is well known as a member of the proinflammatory family, ischemic preconditioning has been shown to enhance IL-6 expression and inhibition of IL-6 could attenuate the early preconditioning effect [119,120]. Importantly, elevation in IL-6 is to some extent responsible for the RIC-mediated cardioprotective effects [121]. Cai et al. demonstrated for the first time that IL-10 upregulation promoted late protection by RIC, possibly through the Stat3 signaling pathway [122]. A more recent study investigated the effects of RIC on certain inflammatory/anti-inflammatory mediator profiles and immune cells, together with the mechanism underlying RIC-mediated neuroprotection [123]. They found that RIC ameliorated the post-stroke reduction of peripheral blood CD3(+)CD8(+) T cells and CD3(+)/CD161a(+) NKT cells markedly, and robustly increased the percentage of CD43(+)/CD172a(+) non-inflammatory monocytes. RIC alone without subsequent stroke obviously promoted plasma IL-6 expression without any impact on the concentration of IL-10 and TNF-α. Interestingly, elevated TNF-α expression and further enhanced IL-6 levels were reported in stroke rats subjected to RIC pretreatment. These findings suggest that changes in immune cells populations and cytokines in circulation may be one mechanism contributing to the RIC-mediated neuroprotection. Moreover, it was also demonstrated that the spleen might play a critical role in RIC-mediated alterations in the peripheral immune system and immunomodulation of the splenic response by RIC might create a favorable immune milieu that affects the progression of stroke [124]. However, scant studies have focused on this area, although more work is on the way.

### *Anti-oxidative stress*

An increasing number of researchers have explored the association between RIC and anti-oxidative activity, suggesting that limb RIC could markedly attenuate IRI through downregulating the expression of free radicals while upregulating the expression of antioxidant proteins. For instance, the level of malondialdehyde (MDA), which is frequently used as an indicator to assess the extent of oxidative stress, was significantly reduced following the application of RIC in combination with or without local IC in different animal models, such as cerebral, myocardial, hepatic and renal ischemic models [125–128]. In addition, RIC showed early promise as a protective approach during primary PCI in STEMI patients to enhance the antioxidant potential (increased levels of glutathione peroxidase, superoxide dismutase and total antioxidant capacity) and suppress the increased MDA level [129].

### *Autophagy*

Recently, several investigations have reported that the neuroprotective effects afforded by RIC are closely associated with autophagy activation and the attenuation of mitochondrial injury following cerebral IRI in rat or mice model [130–133]. These findings imply that autophagy exerts a pivotal function in RIC-induced neuroprotection with the possible involvement of AKT/GSK3β, AMPK, and mTOR/p70S6K signaling pathways, and AKT-dependent Bcl-2 phosphorylation. However, in contrast, studies also demonstrated that inhibition of the autophagy process contributed to the protection against cerebral ischemia injury in rats subjected with ischemic post-conditioning alone or combined with RIC [134,135]. The potential function of autophagy in RIC-induced protection needs to be further elucidated.

### *Improvement of endothelial function and vascular remodeling*

Endothelial dysfunction is a common finding in patients with atherosclerotic artery disease, and associated with unfavorable clinical outcomes [136,137]. Studies have shown that repeated RIC stimulus could improve endothelial function in healthy individuals [138,139]. More importantly, in patients undergoing invasive coronary angiography for stable coronary artery disease, long-term, regular RIC has been found to produce improvements in both peripheral and coronary artery function [140,141]. The enhanced endothelial function may be secondary to circulating mediators activated by RIC, such as an increase in endothelial progenitor cells and/or vascular endothelial growth factor (VEGF). Promoting endothelial cells may release tPA and lower the level of PAI in circulation [142]. Other explanations such as reduced oxidative stress and inflammation, and upregulation of endothelial NOS should be considered as well.

Experimental and our pilot clinical studies reveal that chronic RIC is capable of robustly improving cerebral blood flow in chronic cerebral ischemia [143–145]. This amelioration is likely by promoting cerebral angiogenesis, vascular remodeling and collateral formation, as indicated by increased expression of CD31, α-SMA, and VEGF-receptor, as well as the increase in vessels number and volume (3D visualization of cerebrovasculature).

### *Specific circulating mediators for each organ*

**Figure 4:**
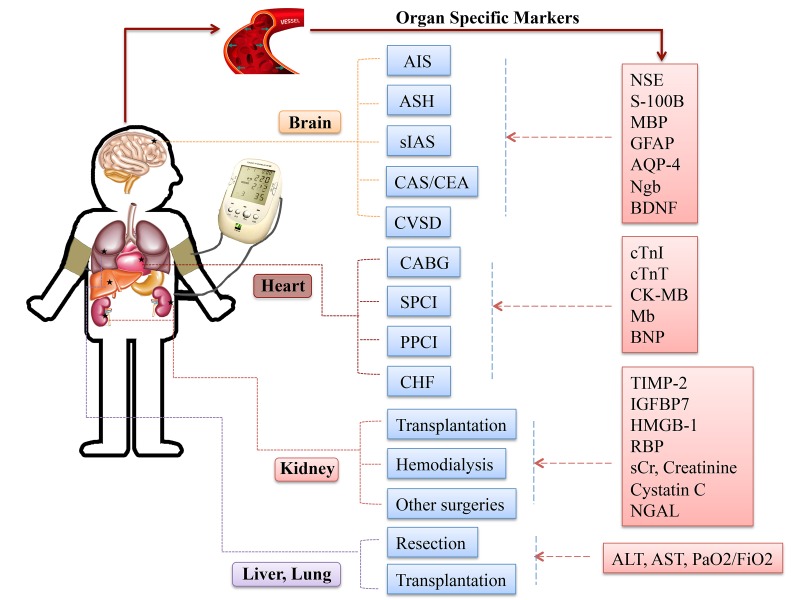
**Specific circulating markers for different organ scenarios.** Abbreviations: AIS, acute ischemic stroke; ASH, acute subarachnoid hemorrhage; sIAS, symptomatic intracranial arterial stenosis; CEA, carotid endarterectomy; CAS, carotid angioplasty and stenting; CVSD, cerebral small vessel disease; CABG, coronary artery bypass graft surgery; SPCI, selective percutaneous coronary intervention; PPCI, primary percutaneous coronary intervention; CHF, chronic heart failure; NSE, neuronal specific enolase; S-100B, S100 calcium binding protein B; GFAP, glial fibrillary acidic protein; AQP-4, aquaporin-4; Ngb, Neuroglobin; cTnI, cardiac troponin I; cTnT, cardiac troponin T; CK-MB, creatine kinase-myocardial band; Mb, myoglobin; BNP, brain natriuretic protein; TIMP-2, tissue inhibitor of metalloproteinases-2; IGFBP-7, insulin like growth factor-binding protein-7; HMGB-1, high-mobility group box protein-1; sCr, serum creatinine; NGAL, neutrophil gelatinase-associated lipocalin; ALT, alanine aminotransferase; AST, aspartate aminotransferase; PaO2/FiO2, partial pressure of oxygen/fraction of inspired oxygen.

Although RIC strategies in different organs/tissues may recruit similar signaling transduction molecules, variations are still recognized, (Fig. 4). Clarifying the role of specific trigger factors for the organ of interest could spur further research in this area and facilitate clinical implement and pharmacological advancement.

### Current clinical evidence of multi-organ protection

**Figure 5:**
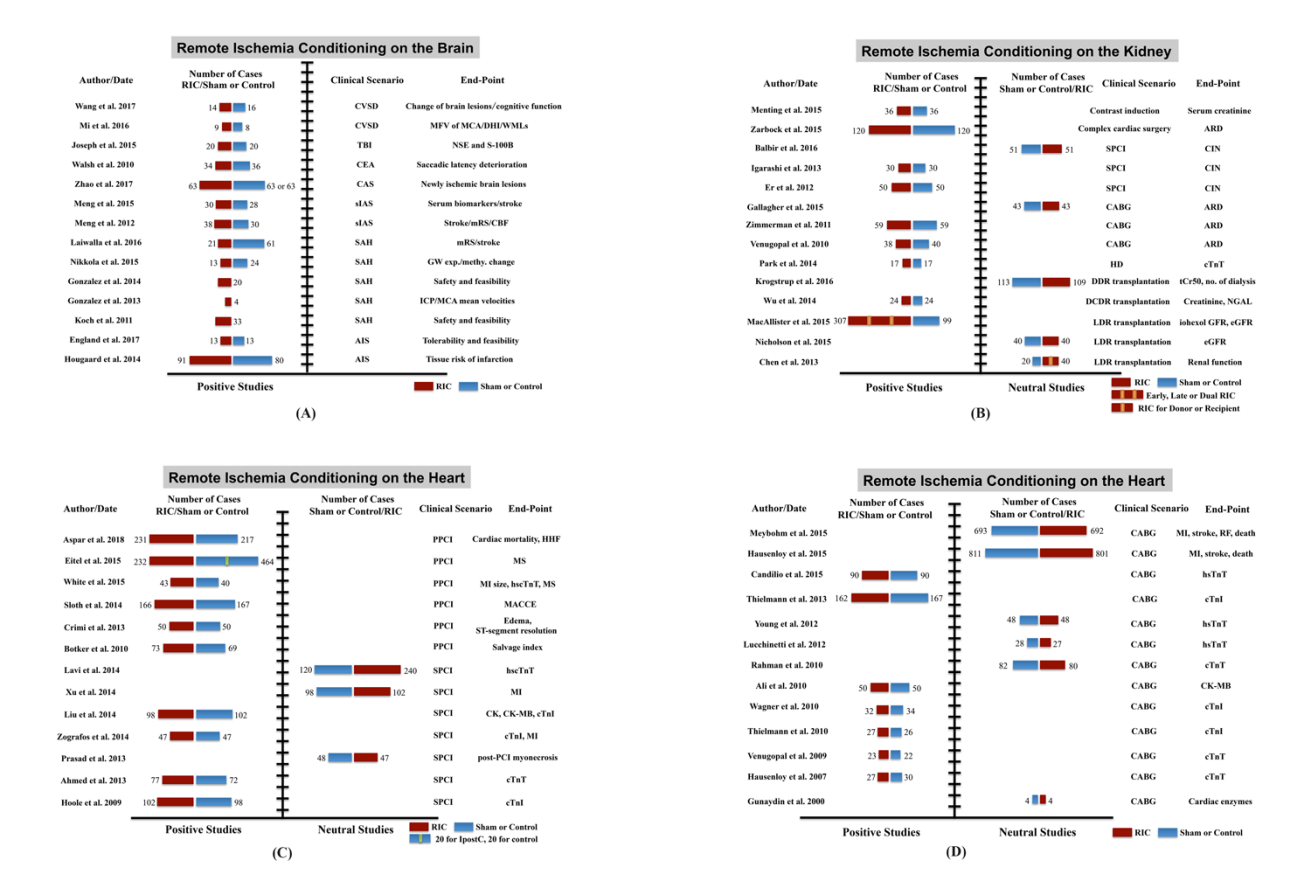
**Clinical trials of remote ischemic conditioning (RIC) on multi-organ protection.** (**A**) Studies of RIC effects on the brain, (**B**) studies of RIC effects on the kidney, (**C**) and (**D**) studies of RIC effects on the heart. Abbreviations: CSVD, cerebral small vessel disease; TBI, traumatic brain injury; CEA, carotid endarterectomy; CAS, carotid angioplasty and stenting; sIAS, symptomatic intracranial arterial stenosis; SAH, subarachnoid hemorrhage; AIS, acute ischemic stroke; MFV, mean flow velocity; MCA, middle cerebral artery; DHI, dizziness handicap inventory; WMLs, white matter lesions; NSE, neuron-specific enolase; S-100B, S100 calcium binding protein B; mRS, modified Rankin scale; CBF, cerebral blood flow; GW exp. and methy. change, genome-wide expression and methylation change; ICP, intracranial pressure; SPCI, selective percutaneous coronary intervention; CABG, coronary artery bypass graft surgery; HD, hemodialysis; DDR, deceased donor renal; DCDR, donation after cardiac death renal; LDR, living-donor renal; ARD, acute renal dysfunction; CIN, contrast-induced nephropathy; tCr50, the estimated time to a 50% decrease in baseline plasma creatinine; NGAL, neutrophil gelatinase-associated lipocalin; eGFR, estimated glomerular filtration rate; PPCI, primary percutaneous coronary intervention; HHF, hospitalization for heart failure; MS, myocardial salvage; MI, myocardial infarction; hs-cTnT, high sensitive-cardiac troponin T; hs-cTnI, high sensitive-cardiac troponin I; MACCE, major adverse cardiac and cerebral event; CK, creatine kinase; CK-MB, creatine kinase-myocardial band; RF, renal failure.

**Figure 6:**
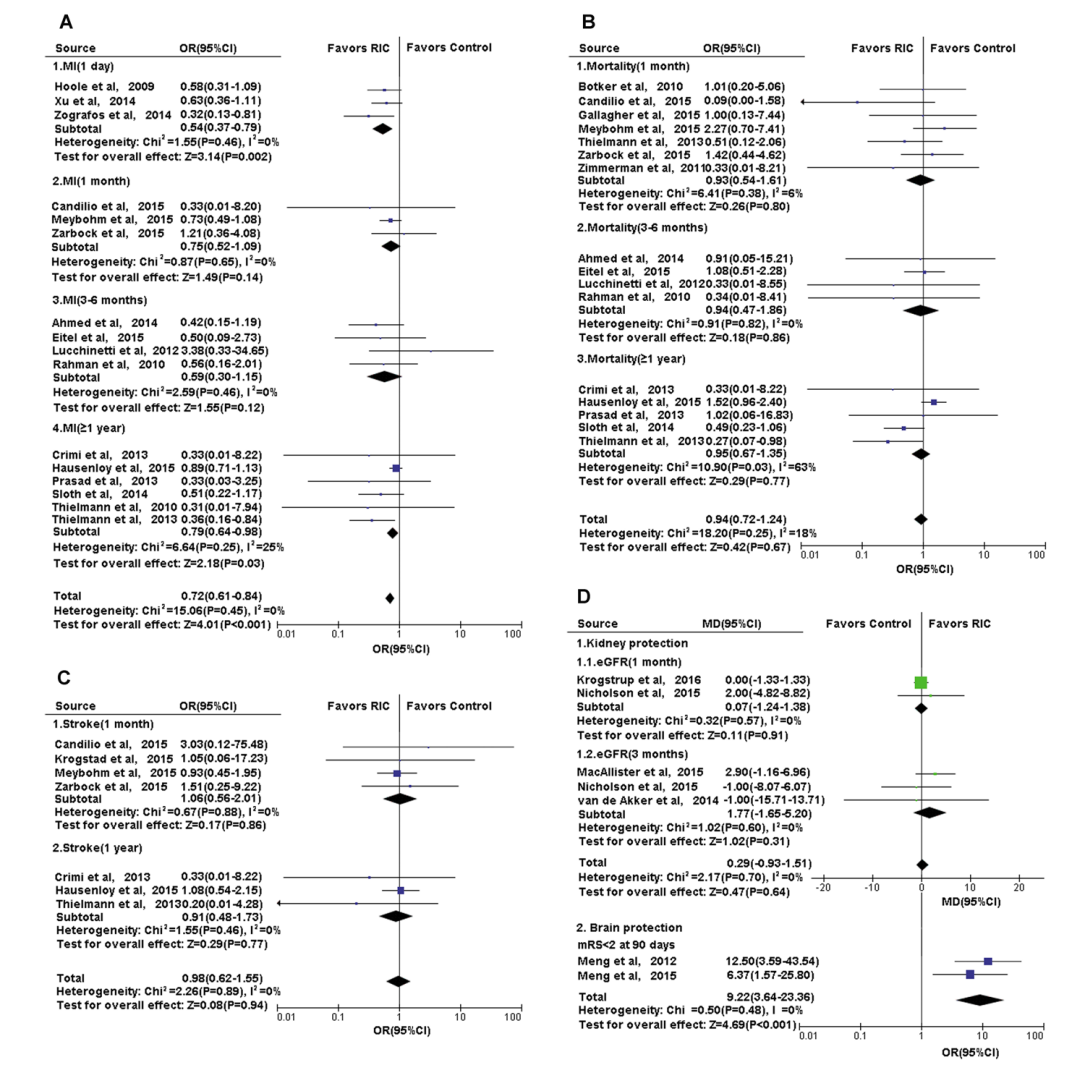
**Forest plot with 95% confidence interval for primary or secondary outcomes.** (**A**) Heart: the incidence of complication MI in recipients who underwent CABG or PCI in RIC group compared with controls; the post-treatment mRS in cerebral infarction treated with RIC compared with controls; (**B**) Heart: the mortality of recipients who underwent CABG or PCI in RIC group compared with controls; (**C**) Heart: the incidence of complication-stroke in recipients who underwent CABG or PCI in RIC group compared with controls; (**D**) Kidney and Brain: the eGFR in recipients who underwent renal transplantation and the post-treatment mRS in cerebral infarction, in RIC group compared with controls. Abbreviations: MI, myocardial infarction; CABG, coronary artery bypass graft surgery; PCI, percutaneous coronary intervention; RIC, remote ischemic conditioning; eGFR, estimated glomerular filtration rate; mRs, modified Rankin scale.

A growing number of clinical trials have implied that RIC is a promising approach to enable multi-organ protection, even though a few studies displayed inconsistent results. Details are shown in Fig. 5 and 6, Supplemental Fig. 1and Supplemental Table 1 to 4.

Evidence collected so far with regard to the scenarios of target organs are listed below.

### *Brain*

(1) Acute ischemic stroke [146,147], RIC treatment after acute stroke was likely to lower the risk of tissue infarction after 1 month, and improve the neurological outcome as depicted by a reduction in day 90 median National Institutes of Health Stroke Scale (NIHSS) score; (2) chronic cerebral ischemia [142,143,148,149], daily use of bilateral upper limb RIC was safe, tolerable, and able to reduce the recurrence of stroke/transient ischemic attack (TIA), improve cerebral perfusion as well as ameliorate the processes of inflammation, coagulation and fibrinolysis; (3) subarachnoid hemorrhage [150–155], the feasibility and tolerability of RIC were denoted in several proof-of-concept trials; (4) carotid endarterectomy and stenting (CEA and CAS) [156,157], a trend toward fewer saccadic latency deteriorations and a significant reduction in the incidence of new DWI lesions were observed among patients who received RIC; (5) cerebral small-vessel disease [158–160], RIC appeared to be effective in retarding cognition decline and decreasing white matter lesions; and (6) traumatic brain injury [161], it was found that RIC markedly reduced the serum levels of neuron-specific enolase (NSE) and S-100β.

### *Heart*

(1) Selective coronary artery bypass surgery (CABG) [140,162–175], the beneficial effect of RIC in patients undergoing CABG surgery has been reported in several proof-of-concept trials, whereas, two recent large prospective, multicenter, double-blinded, randomized, controlled clinical trials yielded negative results, denoting that RIC failed to influence the incidence of major cardiac and cerebral adverse events, myocardial or renal injury biomarkers and quality of life; (2) selective PCI [176–191], the effect of RIC on patients undergoing selective PCI remains uncertain with discrepant results, and a large-scale, multicenter clinical trial is currently ongoing to explore and verify the potential benefits; (3) primary PCI for STEMI [12,14,192–198], data available are in favor of the RIC-mediated advantageous effect, though larger, well-designed and adequately powered multicenter studies are required to confirm the result; and (4) chronic heart failure [199–201], RIC twice daily for a week ameliorated coronary microcirculation in patients with chronic heart failure without eliciting any adverse effects; RIC once daily for four weeks may enhance skeletal muscle function and lower blood pressure in a compensated state of heart failure, while a general improvement of cardiac function was only seen in most severely compromised patients with chronic heart failure; six-week of twice a day RIC could improve cardiac function in patients with stable heart failure.

### *Kidney*

(1) Renal dysfunction after elective coronary revascularization [202–211], in general, data available showed that RIC decreased the acute renal dysfunction incidence in PCI patients but not in CABG, and the overall incidence of renal replacement therapy and mortality reported in CABG subjects were insignificantly rendered by RIC; (2) high risk for acute kidney injury in the setting of cardiac surgery [207,212], RIC markedly reduced the rate of acute kidney injury within the first 3 days after cardiac surgery, and the 3-month incidence of composite major adverse kidney events, including mortality, renal replacement therapy requirement, and renal dysfunction; (3) renal transplantation [213–218], the application of RIC in the kidney transplantation field yielded conflicting results, most of which were neutral; and (4) dialysis-related ischemic injury [219], only one small pilot study demonstrated that RIC prior to each dialysis practice for a month robustly downregulated the cTnT level (at 1 week and 4 weeks) among patients receiving chronic dialysis.

### *Lung*

(1) Pulmonary dysfunction following cardiac surgeries [10,220,221], no significant difference was found in the pulmonary variables such as postoperative partial pressure of oxygen (PaO2)/fraction of inspired oxygen (FiO2) and transpulmonary gradient of inflammatory mediators between the RIC and the control groups, whereas, the incidence of acute lung injury seemed to be reduced by RIC; (2) pulmonary dysfunction following repair of abdominal aortic aneurysm [222], pulmonary dysfunction was robustly attenuated in the RIC patients (higher arterial-alveolar oxygen tension ratio and lower severity of the pulmonary injury score); (3) lung resection for non-small-cell lung cancer [223,224], RIC may afford protection against pulmonary injury as presented by improved intraoperative oxygenation accompanied with the reduced postoperative incidence of acute lung injury; and (4) primary graft dysfunction in the setting of lung transplantation [225], encouraging outcomes were noticed as presented by trends toward a higher level of PaO2/ FiO2, a lower primary graft dysfunction severity score and a reduced incidence of rejection in RIC treated patients.

### *Liver*

(1) Liver injury after cardiac valve replacement surgery [221], the levels of total bilirubin, instead of serum transaminases and albumin, were lower in the RIC group; (2) major liver resection for colorectal liver metastasis [226], RIC significantly ameliorated liver injury after hepatectomy as presented by decreased serum transaminases and higher indocyanine green clearance; and (3) liver transplantation [227,228], a single center, randomized clinical trial aiming to elucidate RIC-mediated protection in liver transplant recipients is underway.

### *Gastrointestinal Organs*

(1) Intestinal injury after open infrarenal abdominal aortic aneurysm repair surgery [222], one small randomized trial assessed the potential use of RIC in GI protection, illustrating that transient RIC substantially reduced the intestinal injury in patients undergoing open infrarenal abdominal aortic aneurysm repair surgery.

### *Skin*

(1) Cutaneous microcirculation in healthy volunteers [229–231], RIC may have a great potential in improving blood perfusion and alleviating soft tissue injury due to accidental or operative trauma and plastic versus reconstructive surgery.

### RIC and ageing

As the average human lifespan has increased markedly over the past decades, the mounting incidence of ageing-related disorders in the elderly, and the related economic and social burden, impel scientists to develop novel strategies to slow down or prevent these disorders, alleviating the suffering at the end of life.

Decreased physiological reserve and tissue resilience are characteristics of biological ageing, which render the human system more susceptible to pathological threats. Given the fact that elderly patients usually have at least two afflicted organs or tissues, therapeutic approaches with systemic actions (inducing protective responses in a wide range of organs and tissues) are warranted. In addition to the efficacy, safety and compliance issues are of great importance in an ageing population. The emerging area of RIC builds upon this foundation. The capability of this non-pharmaceutical and non-surgical intervention to protect vital organs simultaneously by enhancing the body’s powers to adapt to pathological threats could provide a safe, less burdensome, minimally-invasive way for ageing-related disorders. Currently, RIC is being evaluated in a variety of clinical settings such as cerebrovascular disease, coronary artery disease and renal injury that predominantly influence the older population.

### Challenges and promising

To date, regarding the safety and tolerability of the methodology, no RIC-associated adverse events have been reported in the published clinical studies. Although the prospect of clinical transformation of RIC on multi-organ protection is promising, challenges still exist. The controversial findings raise the question “Can RIC have impact on clinical outcomes in patients under different clinical conditions?” To the best of our knowledge, a number of factors should be reconsidered thoroughly in the future work.

For instance, although previous experimental work has implied that the number and duration of IR cycles might affect the efficacy of RIC, there is a paucity of clinical data comparing the effectiveness of different RIC protocols, and no convincing evidence of the most favorable conditioning strategy has been established [232]. Notably, the ideal regimen for limb RIC may vary on the basis of clinical scenarios where they work best. Hence, optimizing the RIC regimens for different clinical settings are pivotal issues waiting to be addressed.

Establishment of biomarkers has been considered as a paramount step for translating RIC from preliminary data into real clinical practice. Many hurdles still lie ahead to screen and discover the appropriate set of biomarkers in order to accurately evaluate the clinical efficacy of RIC. The investigations of a wider panel and combination use of genomics, proteomics and imaging markers are warranted. For instance, it was reported that more than 150 genes including apoptosis, cell survival, and immunity-related genes were differentially expressed in healthy subjects who were preconditioned with repetitive arm ischemia [112]. Following this, a pilot study exploring genome-wide expression and methylation changes in patients with aneurysmal subarachnoid hemorrhage revealed that a group of genes in cell cycle, defense, and inflammatory responses were rendered by RIC [154]. Novel techniques such as gene chip, serial analysis of gene expression and genome-wide expression studies may largely facilitate understanding of the conditioning response [114].

Essentially, the potential applications of RIC are numerous and may be extended far more than aforementioned clinical settings. Fox example, RIC has been found to reduce the severity of acute mountain sickness symptoms, enhance the exercise performance and benefit patients with diabetic foot ulcer [233–237]. On an additional note, animal studies have highlighted the potential of limb RIC in diverse retinal disorders such as promoting retinal ganglion cell survival after optic nerve transection and affording protection to retinal photoreceptors against bright light- or IRI-induced photoreceptor degeneration [238–240]. Knowing that the proposed mechanisms of RIC involve modulation of multiple pathways that cover inflammatory response, apoptosis, and oxidative stress, its therapeutic potential is believed to be able to expand to some non-ischemic inflammatory diseases with at least partially similar pathophysiological processes such as inflammatory bowel disease, acute pancreatitis, and connective tissue disorders. In addition, evidence available suggests that long-term RIC is capable of promoting improvement of the vascular function (such as improvement of local and systemic endothelial function, and microcirculation), which may be related to lower risks of future cerebro- and cardiovascular adverse events [138,139]. As the application of RIC in these probable candidates is still in infancy, the feasibility will be determined in more rigorously designed experimental and clinical studies.

There are conceivably a number of existing and potential stakeholders when it comes to the extrapolation of RIC from test bench to bedside. One of the major explanations for these discrepancies lies in the impact of comorbidities on the ischemic tissues or organs in response to the benefits from IC. Animals used in experimental studies are mainly juvenile, genetically homogenous, housed in similar living environments with the same diets, and carried with limited comorbidities. Nevertheless, a significant challenge in clinical trials is that enrolled individuals are more aged with large comorbidities and polypharmacy. In fact, sets of comorbidities including ageing, hypercholesterolemia, hypertension, diabetes, and chronic kidney disease have been found to inhibit or abolish the potency of IC according to previous experimental findings. It seems that concomitant medications including volatile anesthetics such as propofol and isoflurane, opioids, statins, anti-platelets, ACEI, beta-blockers, nitrates and hypoglycemic agents such as pioglitazone and glimepiride might either abrogate or restore the beneficial effects afforded by IC [167,241–246]. One primary concern in treating patients with acute ischemic cerebro- or cardiovascular accident is the inevitable healthcare transport delay, which is believed to be closely associated with unfavorable clinical outcomes. A single center clinical study reported that RIC conducted during transportation to the hospital lessened the prejudicious effect of transport delay on myocardial salvage in STEMI patients undergoing primary PCI, and this cardioprotective effect was more marked in those with extended delay (healthcare system delay > 120 min) [247]. Moreover, the location and severity of infarcts, and the coronary collateral circulation’s ability may also play a potential role for mediating the effect of RIC [248]. A better realization regarding the limitations of current experimental or clinical design together with the way through which comorbidities, medications and other factors affect the efficacy of RIC will facilitate the selection of beneficial population, help understanding the underlying mechanisms and optimize the RIC regimen.

Intriguingly, pharmacological manipulation targeting the signaling cascades and related molecular participants that can modulate the endogenous mechanisms of RIC is an emerging notion with substantial clinical value. Figuring out common mechanisms of protection afforded by IC and some pharmacological agents in a given clinical set-up may facilitate the development of novel approaches against deleterious effects such as IRI. In addition, the presence of time windows during which the signaling cascades in responsive to IRI are engaged might affect not only the effectiveness of IC but medical interventions that target perpetrators of reperfusion injury. Clearly, research should be continue for the sake of identifying pharmacological mimetic candidates that are safe and efficacious, verifying time window for conditioning, and further delineating the mechanism behind as well as human genetic and proteomic responses to conditioning.

### Summary and future direction

RIC is an innovative therapeutic strategy, whereby transient, repetitive, sublethal ischemic exposure to a particular tissue or organ is able to confer systemic protection to distant susceptible organs or tissues against subsequent IRI.

The application of RIC is simple and non-invasive, and has been extended from the arena of initial cardiac protection to a number of other organs such as the brain, kidney, lung and liver. The feature of multi-organ protection is quite significant since it allows RIC to a variety of clinical settings in which organs or tissues are vulnerable to acute IRI. It is hopeful that future well-designed clinical trials with adequate size and power will turn RIC into a routine clinical practice for either prevention or attenuation of disorders in more extensive scenarios than currently expected.

### Key points

In this study, we provided an overview of the basic concept and proposed protective mechanisms of remote ischemic conditioning (RIC). In addition, we comprehensively reviewed and summarized the clinical studies of RIC in multi-organ protection published so far. Potential obstacles, which may retard the clinical translation of RIC, were discussed as well. We found that:

Proof-of-concept clinical studies demonstrated benefits with RIC as an adjuvant to thrombolysis, mechanical thrombectomy or primary angioplasty in patients with acute cerebro- or cardiovascular attack, such as ischemic stroke and acute myocardial infarction; in light of advantages including simplicity, low expenditure and safety, RIC may be an ideal intervention to be employed during the transportation to qualified stroke or chest pain centers, and before and/or after intrahospital treatment.

Long-term, repeated use of RIC has the potential to improve vascular endothelial function and enhance tissue perfusion, whereby decreasing the incidence or recurrence of unfavorable clinical endpoints in diverse clinical settings, such as chronic cerebral ischemia and chronic heart failure.

Although two large, multicenter, randomized clinical trials of RIC in coronary artery bypass graft surgery yielded negative outcomes, factors including patient selection, RIC protocol and potential confounders should be further explored before reaching a final conclusion.

Regarding the multi-organ protection feature, RIC seems to be a promising non-pharmaceutical and non-surgical approach for preventing and treating systemic vascular diseases, which can inflict multiple systems simultaneously. As multi-organ ischemia-reperfusion injury, particularly amongst elderly patients with clinical scenarios such as atherosclerotic vascular disease, heart surgery and organ transplantation, is one of the most common real-world situations that besets clinicians, the authors believe that this paper will certainly arouse a wide interest from medical workers in both tertiary and community-based medical facilities.

Future experimental or clinical work should focus on addressing the issues that may influence the translation of RIC from test bench to bedside, such as identifying the protective mechanism underlying ischemic conditioning, optimizing the conditioning regimen, establishing biomarkers to accurately evaluate the efficacy of RIC, and figuring out the impact of potential comorbidities, medications and other factors on RIC.

## METHODS

We searched PubMed and EMBASE to identify randomized controlled trials (RCTs), meta-analyses, observational studies and systematic reviews that published between 1986 and 2018, via key words of ‘remote ischemic conditioning’, ‘remote ischemic preconditioning’, ‘remote ischemic perconditioning’, ‘remote ischemic postconditioning’ etc. We subsequently reviewed references of the retrieved articles for additional reports. Meta-analyses and systematic reviews were prioritized; case series and reports were included only for interventions for which RCTs were not available. Some data from prospective clinical trials were processed using RevMan5.0 software provided by Cochrane collaboration and transformed via relevant formulas.

## SUPPLEMENTARY MATERIAL

Supplemental Figure 1

Supplemental Table 1-4

## References

[1] Schmidt MR, Pryds K, Bøtker HE. Novel adjunctive treatments of myocardial infarction. World J Cardiol. 2014; 6:434–43. 10.4330/wjc.v6.i6.434 24976915 PMC4072833

[2] Yellon DM, Hausenloy DJ. Myocardial reperfusion injury. N Engl J Med. 2007; 357:1121–35. 10.1056/NEJMra071667 17855673

[3] Hess DC, Blauenfeldt RA, Andersen G, Hougaard KD, Hoda MN, Ding Y, Ji X. Remote ischaemic conditioning-a new paradigm of self-protection in the brain. Nat Rev Neurol. 2015; 11:698–710. 10.1038/nrneurol.2015.223 26585977

[4] Murry CE, Jennings RB, Reimer KA. Preconditioning with ischemia: a delay of lethal cell injury in ischemic myocardium. Circulation. 1986; 74:1124–36. 10.1161/01.CIR.74.5.1124 3769170

[5] Walsh SR, Tang TY, Kullar P, Jenkins DP, Dutka DP, Gaunt ME. Ischaemic preconditioning during cardiac surgery: systematic review and meta-analysis of perioperative outcomes in randomised clinical trials. Eur J Cardiothorac Surg. 2008; 34:985–94. 10.1016/j.ejcts.2008.07.062 18783958

[6] Przyklenk K, Bauer B, Ovize M, Kloner RA, Whittaker P. Regional ischemic ‘preconditioning’ protects remote virgin myocardium from subsequent sustained coronary occlusion. Circulation. 1993; 87:893–99. 10.1161/01.CIR.87.3.893 7680290

[7] Gho BC, Schoemaker RG, van den Doel MA, Duncker DJ, Verdouw PD. Myocardial protection by brief ischemia in noncardiac tissue. Circulation. 1996; 94:2193–200. 10.1161/01.CIR.94.9.2193 8901671

[8] Birnbaum Y, Hale SL, Kloner RA. Ischemic preconditioning at a distance: reduction of myocardial infarct size by partial reduction of blood supply combined with rapid stimulation of the gastrocnemius muscle in the rabbit. Circulation. 1997; 96:1641–46. 10.1161/01.CIR.96.5.1641 9315559

[9] Kharbanda RK, Mortensen UM, White PA, Kristiansen SB, Schmidt MR, Hoschtitzky JA, Vogel M, Sorensen K, Redington AN, MacAllister R. Transient limb ischemia induces remote ischemic preconditioning in vivo. Circulation. 2002; 106:2881–83. 10.1161/01.CIR.0000043806.51912.9B 12460865

[10] Cheung MM, Kharbanda RK, Konstantinov IE, Shimizu M, Frndova H, Li J, Holtby HM, Cox PN, Smallhorn JF, Van Arsdell GS, Redington AN. Randomized controlled trial of the effects of remote ischemic preconditioning on children undergoing cardiac surgery: first clinical application in humans. J Am Coll Cardiol. 2006; 47:2277–82. 10.1016/j.jacc.2006.01.066 16750696

[11] Schmidt MR, Smerup M, Konstantinov IE, Shimizu M, Li J, Cheung M, White PA, Kristiansen SB, Sorensen K, Dzavik V, Redington AN, Kharbanda RK. Intermittent peripheral tissue ischemia during coronary ischemia reduces myocardial infarction through a KATP-dependent mechanism: first demonstration of remote ischemic perconditioning. Am J Physiol Heart Circ Physiol. 2007; 292:H1883–90. 10.1152/ajpheart.00617.2006 17172279

[12] Rentoukas I, Giannopoulos G, Kaoukis A, Kossyvakis C, Raisakis K, Driva M, Panagopoulou V, Tsarouchas K, Vavetsi S, Pyrgakis V, Deftereos S. Cardioprotective role of remote ischemic periconditioning in primary percutaneous coronary intervention: enhancement by opioid action. JACC Cardiovasc Interv. 2010; 3:49–55. 10.1016/j.jcin.2009.10.015 20129568

[13] Li L, Luo W, Huang L, Zhang W, Gao Y, Jiang H, Zhang C, Long L, Chen S. Remote perconditioning reduces myocardial injury in adult valve replacement: a randomized controlled trial. J Surg Res. 2010; 164:e21–26. 10.1016/j.jss.2010.06.016 20850778

[14] Bøtker HE, Kharbanda R, Schmidt MR, Bøttcher M, Kaltoft AK, Terkelsen CJ, Munk K, Andersen NH, Hansen TM, Trautner S, Lassen JF, Christiansen EH, Krusell LR, et al. Remote ischaemic conditioning before hospital admission, as a complement to angioplasty, and effect on myocardial salvage in patients with acute myocardial infarction: a randomised trial. Lancet. 2010; 375:727–34. 10.1016/S0140-6736(09)62001-8 20189026

[15] Zhao ZQ, Corvera JS, Halkos ME, Kerendi F, Wang NP, Guyton RA, Vinten-Johansen J. Inhibition of myocardial injury by ischemic postconditioning during reperfusion: comparison with ischemic preconditioning. Am J Physiol Heart Circ Physiol. 2003; 285:H579–88. 10.1152/ajpheart.01064.2002 12860564

[16] Staat P, Rioufol G, Piot C, Cottin Y, Cung TT, L’Huillier I, Aupetit JF, Bonnefoy E, Finet G, André-Fouët X, Ovize M. Postconditioning the human heart. Circulation. 2005; 112:2143–48. 10.1161/CIRCULATIONAHA.105.558122 16186417

[17] Li B, Chen R, Huang R, Luo W. Clinical benefit of cardiac ischemic postconditioning in corrections of tetralogy of Fallot. Interact Cardiovasc Thorac Surg. 2009; 8:17–21. 10.1510/icvts.2008.189373 18854339

[18] Luo W, Li B, Chen R, Huang R, Lin G. Effect of ischemic postconditioning in adult valve replacement. Eur J Cardiothorac Surg. 2008; 33:203–08. 10.1016/j.ejcts.2007.11.010 18078762

[19] Lønborg J, Kelbaek H, Vejlstrup N, Jørgensen E, Helqvist S, Saunamäki K, Clemmensen P, Holmvang L, Treiman M, Jensen JS, Engstrøm T. Cardioprotective effects of ischemic postconditioning in patients treated with primary percutaneous coronary intervention, evaluated by magnetic resonance. Circ Cardiovasc Interv. 2010; 3:34–41. 10.1161/CIRCINTERVENTIONS.109.905521 20118154

[20] Gao J, Luo J, Liu F, Zheng Y, Chen B, Chen Q, Yang Y. Short-and long-term effects of ischemic postconditioning in STEMI patients: a meta-analysis. Lipids Health Dis. 2015; 14:147. 10.1186/s12944-015-0151-x 26573572 PMC4647593

[21] Freixa X, Bellera N, Ortiz-Pérez JT, Jiménez M, Paré C, Bosch X, De Caralt TM, Betriu A, Masotti M. Ischaemic postconditioning revisited: lack of effects on infarct size following primary percutaneous coronary intervention. Eur Heart J. 2012; 33:103–12. 10.1093/eurheartj/ehr297 21846677

[22] Tarantini G, Favaretto E, Marra MP, Frigo AC, Napodano M, Cacciavillani L, Giovagnoni A, Renda P, De Biasio V, Plebani M, Mion M, Zaninotto M, Isabella G, et al. Postconditioning during coronary angioplasty in acute myocardial infarction: the POST-AMI trial. Int J Cardiol. 2012; 162:33–38. 10.1016/j.ijcard.2012.03.136 22494866

[23] Engstrøm T, Kelbæk H, Helqvist S, Høfsten DE, Kløvgaard L, Clemmensen P, Holmvang L, Jørgensen E, Pedersen F, Saunamaki K, Ravkilde J, Tilsted HH, Villadsen A, et al, and Third Danish Study of Optimal Acute Treatment of Patients With ST Elevation Myocardial Infarction–Ischemic Postconditioning (DANAMI-3–iPOST) Investigators. Effect of ischemic postconditioning during primary percutaneous coronary intervention for patients with ST-segment elevation myocardial infarction: a randomized clinical trial. JAMA Cardiol. 2017; 2:490–97. 10.1001/jamacardio.2017.0022 28249094 PMC5814983

[24] Marber MS, Latchman DS, Walker JM, Yellon DM. Cardiac stress protein elevation 24 hours after brief ischemia or heat stress is associated with resistance to myocardial infarction. Circulation. 1993; 88:1264–72. 10.1161/01.CIR.88.3.1264 8353888

[25] Kuzuya T, Hoshida S, Yamashita N, Fuji H, Oe H, Hori M, Kamada T, Tada M. Delayed effects of sublethal ischemia on the acquisition of tolerance to ischemia. Circ Res. 1993; 72:1293–99. 10.1161/01.RES.72.6.1293 8495557

[26] Guo Y, Wu WJ, Qiu Y, Tang XL, Yang Z, Bolli R. Demonstration of an early and a late phase of ischemic preconditioning in mice. Am J Physiol. 1998; 275:H1375–87. 9746488 10.1152/ajpheart.1998.275.4.H1375 PMC3701297

[27] Malhotra S, Naggar I, Stewart M, Rosenbaum DM. Neurogenic pathway mediated remote preconditioning protects the brain from transient focal ischemic injury. Brain Res. 2011; 1386:184–90. 10.1016/j.brainres.2011.02.032 21338588

[28] Saxena P, Newman MA, Shehatha JS, Redington AN, Konstantinov IE. Remote ischemic conditioning: evolution of the concept, mechanisms, and clinical application. J Card Surg. 2010; 25:127–34. 10.1111/j.1540-8191.2009.00820.x 19549044

[29] Pan J, Li X, Peng Y. Remote ischemic conditioning for acute ischemic stroke: dawn in the darkness. Rev Neurosci. 2016; 27:501–10. 10.1515/revneuro-2015-0043 26812782

[30] Hausenloy DJ, Yellon DM. The second window of preconditioning (SWOP) where are we now? Cardiovasc Drugs Ther. 2010; 24:235–54. 10.1007/s10557-010-6237-9 20496105

[31] Dezfulian C, Garrett M, Gonzalez NR. Clinical application of preconditioning and postconditioning to achieve neuroprotection. Transl Stroke Res. 2013; 4:19–24. 10.1007/s12975-012-0224-3 24323188 PMC4224593

[32] Ren C, Gao X, Niu G, Yan Z, Chen X, Zhao H. Delayed postconditioning protects against focal ischemic brain injury in rats. PLoS One. 2008; 3:e3851. 10.1371/journal.pone.0003851 19066627 PMC2588536

[33] Sun J, Tong L, Luan Q, Deng J, Li Y, Li Z, Dong H, Xiong L. Protective effect of delayed remote limb ischemic postconditioning: role of mitochondrial K(ATP) channels in a rat model of focal cerebral ischemic reperfusion injury. J Cereb Blood Flow Metab. 2012; 32:851–59. 10.1038/jcbfm.2011.199 22274742 PMC3345910

[34] Loukogeorgakis SP, Panagiotidou AT, Broadhead MW, Donald A, Deanfield JE, MacAllister RJ. Remote ischemic preconditioning provides early and late protection against endothelial ischemia-reperfusion injury in humans: role of the autonomic nervous system. J Am Coll Cardiol. 2005; 46:450–56. 10.1016/j.jacc.2005.04.044 16053957

[35] Donato M, Buchholz B, Rodríguez M, Pérez V, Inserte J, García-Dorado D, Gelpi RJ. Role of the parasympathetic nervous system in cardioprotection by remote hindlimb ischaemic preconditioning. Exp Physiol. 2013; 98:425–34. 10.1113/expphysiol.2012.066217 22872660

[36] Gill R, Kuriakose R, Gertz ZM, Salloum FN, Xi L, Kukreja RC. Remote ischemic preconditioning for myocardial protection: update on mechanisms and clinical relevance. Mol Cell Biochem. 2015; 402:41–49. 10.1007/s11010-014-2312-z 25552250

[37] Ren C, Yan Z, Wei D, Gao X, Chen X, Zhao H. Limb remote ischemic postconditioning protects against focal ischemia in rats. Brain Res. 2009; 1288:88–94. 10.1016/j.brainres.2009.07.029 19631625 PMC2744502

[38] Brzozowski T, Konturek PC, Pajdo R, Kwiecień S, Sliwowski Z, Drozdowicz D, Ptak-Belowska A, Pawlik M, Konturek SJ, Pawlik WW, Hahn GG. Importance of brain-gut axis in the gastroprotection induced by gastric and remote preconditioning. J Physiol Pharmacol. 2004; 55:165–77. 15082876

[39] Xiao L, Lu R, Hu CP, Deng HW, Li YJ. Delayed cardioprotection by intestinal preconditioning is mediated by calcitonin gene-related peptide. Eur J Pharmacol. 2001; 427:131–35. 10.1016/S0014-2999(01)01231-6 11557265

[40] Wong GT, Lu Y, Mei B, Xia Z, Irwin MG. Cardioprotection from remote preconditioning involves spinal opioid receptor activation. Life Sci. 2012; 91:860–65. 10.1016/j.lfs.2012.08.037 22982345

[41] Basalay M, Barsukevich V, Mastitskaya S, Mrochek A, Pernow J, Sjöquist PO, Ackland GL, Gourine AV, Gourine A. Remote ischaemic pre- and delayed postconditioning - similar degree of cardioprotection but distinct mechanisms. Exp Physiol. 2012; 97:908–17. 10.1113/expphysiol.2012.064923 22427438 PMC3470925

[42] Mastitskaya S, Marina N, Gourine A, Gilbey MP, Spyer KM, Teschemacher AG, Kasparov S, Trapp S, Ackland GL, Gourine AV. Cardioprotection evoked by remote ischaemic preconditioning is critically dependent on the activity of vagal pre-ganglionic neurones. Cardiovasc Res. 2012; 95:487–94. 10.1093/cvr/cvs212 22739118 PMC3422080

[43] Henninger N, Fisher M. Stimulating circle of Willis nerve fibers preserves the diffusion-perfusion mismatch in experimental stroke. Stroke. 2007; 38:2779–86. 10.1161/STROKEAHA.107.485581 17761922

[44] Sun Z, Baker W, Hiraki T, Greenberg JH. The effect of right vagus nerve stimulation on focal cerebral ischemia: an experimental study in the rat. Brain Stimul. 2012; 5:1–10. 10.1016/j.brs.2011.01.009 22037134 PMC3264742

[45] Enko K, Nakamura K, Yunoki K, Miyoshi T, Akagi S, Yoshida M, Toh N, Sangawa M, Nishii N, Nagase S, Kohno K, Morita H, Kusano KF, Ito H. Intermittent arm ischemia induces vasodilatation of the contralateral upper limb. J Physiol Sci. 2011; 61:507–13. 10.1007/s12576-011-0172-9 21901641 PMC10718035

[46] Heusch G, Bøtker HE, Przyklenk K, Redington A, Yellon D. Remote ischemic conditioning. J Am Coll Cardiol. 2015; 65:177–95. 10.1016/j.jacc.2014.10.031 25593060 PMC4297315

[47] Dickson EW, Reinhardt CP, Renzi FP, Becker RC, Porcaro WA, Heard SO. Ischemic preconditioning may be transferable via whole blood transfusion: preliminary evidence. J Thromb Thrombolysis. 1999; 8:123–29. 10.1023/A:1008911101951 10436142

[48] Konstantinov IE, Li J, Cheung MM, Shimizu M, Stokoe J, Kharbanda RK, Redington AN. Remote ischemic preconditioning of the recipient reduces myocardial ischemia-reperfusion injury of the denervated donor heart via a Katp channel-dependent mechanism. Transplantation. 2005; 79:1691–95. 10.1097/01.TP.0000159137.76400.5D 15973170

[49] Helgeland E, Breivik LE, Vaudel M, Svendsen OS, Garberg H, Nordrehaug JE, Berven FS, Jonassen AK. Exploring the human plasma proteome for humoral mediators of remote ischemic preconditioning--a word of caution. PLoS One. 2014; 9:e109279. 10.1371/journal.pone.0109279 25333471 PMC4198105

[50] Lim SY, Yellon DM, Hausenloy DJ. The neural and humoral pathways in remote limb ischemic preconditioning. Basic Res Cardiol. 2010; 105:651–55. 10.1007/s00395-010-0099-y 20449597

[51] Jensen RV, Støttrup NB, Kristiansen SB, Bøtker HE. Release of a humoral circulating cardioprotective factor by remote ischemic preconditioning is dependent on preserved neural pathways in diabetic patients. Basic Res Cardiol. 2012; 107:285. 10.1007/s00395-012-0285-1 22821347

[52] Mastitskaya S, Basalay M, Hosford PS, Ramage AG, Gourine A, Gourine AV. Identifying the source of a humoral factor of remote (pre)conditioning cardioprotection. PLoS One. 2016; 11:e0150108. 10.1371/journal.pone.0150108 26918777 PMC4769182

[53] Cai Z, Manalo DJ, Wei G, Rodriguez ER, Fox-Talbot K, Lu H, Zweier JL, Semenza GL. Hearts from rodents exposed to intermittent hypoxia or erythropoietin are protected against ischemia-reperfusion injury. Circulation. 2003; 108:79–85. 10.1161/01.CIR.0000078635.89229.8A 12796124

[54] Cai Z, Zhong H, Bosch-Marce M, Fox-Talbot K, Wang L, Wei C, Trush MA, Semenza GL. Complete loss of ischaemic preconditioning-induced cardioprotection in mice with partial deficiency of HIF-1 alpha. Cardiovasc Res. 2008; 77:463–70. 10.1093/cvr/cvm035 18006459

[55] Eckle T, Köhler D, Lehmann R, El Kasmi K, Eltzschig HK. Hypoxia-inducible factor-1 is central to cardioprotection: a new paradigm for ischemic preconditioning. Circulation. 2008; 118:166–75. 10.1161/CIRCULATIONAHA.107.758516 18591435

[56] Kant R, Diwan V, Jaggi AS, Singh N, Singh D. Remote renal preconditioning-induced cardioprotection: a key role of hypoxia inducible factor-prolyl 4-hydroxylases. Mol Cell Biochem. 2008; 312:25–31. 10.1007/s11010-008-9717-5 18273560

[57] Cai Z, Luo W, Zhan H, Semenza GL. Hypoxia-inducible factor 1 is required for remote ischemic preconditioning of the heart. Proc Natl Acad Sci USA. 2013; 110:17462–67. 10.1073/pnas.1317158110 24101519 PMC3808664

[58] Kalakech H, Tamareille S, Pons S, Godin-Ribuot D, Carmeliet P, Furber A, Martin V, Berdeaux A, Ghaleh B, Prunier F. Role of hypoxia inducible factor-1α in remote limb ischemic preconditioning. J Mol Cell Cardiol. 2013; 65:98–104. 10.1016/j.yjmcc.2013.10.001 24140799

[59] Albrecht M, Zitta K, Bein B, Wennemuth G, Broch O, Renner J, Schuett T, Lauer F, Maahs D, Hummitzsch L, Cremer J, Zacharowski K, Meybohm P. Remote ischemic preconditioning regulates HIF-1α levels, apoptosis and inflammation in heart tissue of cardiosurgical patients: a pilot experimental study. Basic Res Cardiol. 2013; 108:314. 10.1007/s00395-012-0314-0 23203207

[60] Xia M, Ding Q, Zhang Z, Feng Q. Remote limb ischemic preconditioning protects rats against cerebral ischemia via HIF-1alpha/AMPK/HSP70 pathway. Cell Mol Neurobiol. 2017; 37:1105–14. 10.1007/s10571-016-0444-2 27896629 PMC11482205

[61] Schwanke U, Konietzka I, Duschin A, Li X, Schulz R, Heusch G. No ischemic preconditioning in heterozygous connexin43-deficient mice. Am J Physiol Heart Circ Physiol. 2002; 283:H1740–42. 10.1152/ajpheart.00442.2002 12234831

[62] Schwanke U, Li X, Schulz R, Heusch G. No ischemic preconditioning in heterozygous connexin 43-deficient mice--a further in vivo study. Basic Res Cardiol. 2003; 98:181–82. 12892084 10.1007/s003950300002

[63] Sánchez JA, Rodríguez-Sinovas A, Barba I, Miró-Casas E, Fernández-Sanz C, Ruiz-Meana M, Alburquerque-Béjar JJ, García-Dorado D. Activation of RISK and SAFE pathways is not involved in the effects of Cx43 deficiency on tolerance to ischemia-reperfusion injury and preconditioning protection. Basic Res Cardiol. 2013; 108:351. 10.1007/s00395-013-0351-3 23595215

[64] Brandenburger T, Huhn R, Galas A, Pannen BH, Keitel V, Barthel F, Bauer I, Heinen A. Remote ischemic preconditioning preserves Connexin 43 phosphorylation in the rat heart in vivo. J Transl Med. 2014; 12:228. 10.1186/s12967-014-0228-8 25159820 PMC4256705

[65] Zhang X, Wang X, Zhu H, Zhu C, Wang Y, Pu WT, Jegga AG, Fan GC. Synergistic effects of the GATA-4-mediated miR-144/451 cluster in protection against simulated ischemia/reperfusion-induced cardiomyocyte death. J Mol Cell Cardiol. 2010; 49:841–50. 10.1016/j.yjmcc.2010.08.007 20708014 PMC2949485

[66] Wang X, Zhu H, Zhang X, Liu Y, Chen J, Medvedovic M, Li H, Weiss MJ, Ren X, Fan GC. Loss of the miR-144/451 cluster impairs ischaemic preconditioning-mediated cardioprotection by targeting Rac-1. Cardiovasc Res. 2012; 94:379–90. 10.1093/cvr/cvs096 22354898 PMC3331614

[67] Varga ZV, Zvara A, Faragó N, Kocsis GF, Pipicz M, Gáspár R, Bencsik P, Görbe A, Csonka C, Puskás LG, Thum T, Csont T, Ferdinandy P. MicroRNAs associated with ischemia-reperfusion injury and cardioprotection by ischemic pre- and postconditioning: protectomiRs. Am J Physiol Heart Circ Physiol. 2014; 307:H216–27. 10.1152/ajpheart.00812.2013 24858849

[68] Li J, Rohailla S, Gelber N, Rutka J, Sabah N, Gladstone RA, Wei C, Hu P, Kharbanda RK, Redington AN. MicroRNA-144 is a circulating effector of remote ischemic preconditioning. Basic Res Cardiol. 2014; 109:423. 10.1007/s00395-014-0423-z 25060662

[69] Brandenburger T, Grievink H, Heinen N, Barthel F, Huhn R, Stachuletz F, Kohns M, Pannen B, Bauer I. Effects of remote ischemic preconditioning and myocardial ischemia on microRNA-1 expression in the rat heart in vivo. Shock. 2014; 42:234–38. 10.1097/SHK.0000000000000201 24978894

[70] Lu Y, Zhang Y, Shan H, Pan Z, Li X, Li B, Xu C, Zhang B, Zhang F, Dong D, Song W, Qiao G, Yang B. MicroRNA-1 downregulation by propranolol in a rat model of myocardial infarction: a new mechanism for ischaemic cardioprotection. Cardiovasc Res. 2009; 84:434–41. 10.1093/cvr/cvp232 19581315

[71] Lee ST, Chu K, Jung KH, Yoon HJ, Jeon D, Kang KM, Park KH, Bae EK, Kim M, Lee SK, Roh JK. MicroRNAs induced during ischemic preconditioning. Stroke. 2010; 41:1646–51. 10.1161/STROKEAHA.110.579649 20576953

[72] Lee YJ, Johnson KR, Hallenbeck JM. Global protein conjugation by ubiquitin-like-modifiers during ischemic stress is regulated by microRNAs and confers robust tolerance to ischemia. PLoS One. 2012; 7:e47787. 10.1371/journal.pone.0047787 23094087 PMC3475703

[73] Dharap A, Vemuganti R. Ischemic pre-conditioning alters cerebral microRNAs that are upstream to neuroprotective signaling pathways. J Neurochem. 2010; 113:1685–91. 20402966 10.1111/j.1471-4159.2010.06735.x PMC2919749

[74] Lusardi TA, Farr CD, Faulkner CL, Pignataro G, Yang T, Lan J, Simon RP, Saugstad JA. Ischemic preconditioning regulates expression of microRNAs and a predicted target, MeCP2, in mouse cortex. J Cereb Blood Flow Metab. 2010; 30:744–56. 10.1038/jcbfm.2009.253 20010955 PMC2935903

[75] Pryds K, Nielsen RR, Hoff CM, Tolbod LP, Bouchelouche K, Li J, Schmidt MR, Redington AN, Frøkiær J, Bøtker HE. Effect of remote ischemic conditioning on myocardial perfusion in patients with suspected ischemic coronary artery disease. J Nucl Cardiol. 2018; 25:887–96. 10.1007/s12350-016-0709-7 27804070

[76] Rassaf T, Totzeck M, Hendgen-Cotta UB, Shiva S, Heusch G, Kelm M. Circulating nitrite contributes to cardioprotection by remote ischemic preconditioning. Circ Res. 2014; 114:1601–10. 10.1161/CIRCRESAHA.114.303822 24643960

[77] Corti P, Gladwin MT. Is nitrite the circulating endocrine effector of remote ischemic preconditioning? Circ Res. 2014; 114:1554–57. 10.1161/CIRCRESAHA.114.303960 24812347 PMC4073608

[78] Siddiqi N, Neil C, Bruce M, MacLennan G, Cotton S, Papadopoulou S, Feelisch M, Bunce N, Lim PO, Hildick-Smith D, Horowitz J, Madhani M, Boon N, et al, and NIAMI investigators. Intravenous sodium nitrite in acute ST-elevation myocardial infarction: a randomized controlled trial (NIAMI). Eur Heart J. 2014; 35:1255–62. 10.1093/eurheartj/ehu096 24639423 PMC4019912

[79] Arroyo-Martínez EA, Meaney A, Gutiérrez-Salmeán G, Rivera-Capello JM, González-Coronado V, Alcocer-Chauvet A, Castillo G, Nájera N, Ceballos G, Meaney E. Is local nitric oxide availability responsible for myocardial salvage after remote preconditioning? Arq Bras Cardiol. 2016; 107:154–62. 27411096 10.5935/abc.20160100 PMC5074068

[80] Zhao HG, Sun XC, Xian XH, Li WB, Zhang M, Li QJ. The role of nitric oxide in the neuroprotection of limb ischemic preconditioning in rats. Neurochem Res. 2007; 32:1919–26. 10.1007/s11064-007-9381-2 17551830

[81] Peng B, Guo QL, He ZJ, Ye Z, Yuan YJ, Wang N, Zhou J. Remote ischemic postconditioning protects the brain from global cerebral ischemia/reperfusion injury by up-regulating endothelial nitric oxide synthase through the PI3K/Akt pathway. Brain Res. 2012; 1445:92–102. 10.1016/j.brainres.2012.01.033 22325092

[82] Pignataro G, Esposito E, Sirabella R, Vinciguerra A, Cuomo O, Di Renzo G, Annunziato L. nNOS and p-ERK involvement in the neuroprotection exerted by remote postconditioning in rats subjected to transient middle cerebral artery occlusion. Neurobiol Dis. 2013; 54:105–14. 10.1016/j.nbd.2013.02.008 23454199

[83] Davidson SM, Selvaraj P, He D, Boi-Doku C, Yellon RL, Vicencio JM, Yellon DM. Remote ischaemic preconditioning involves signalling through the SDF-1α/CXCR4 signalling axis. Basic Res Cardiol. 2013; 108:377. 10.1007/s00395-013-0377-6 23917520

[84] Jiang Q, Song P, Wang E, Li J, Hu S, Zhang H. Remote ischemic postconditioning enhances cell retention in the myocardium after intravenous administration of bone marrow mesenchymal stromal cells. J Mol Cell Cardiol. 2013; 56:1–7. 10.1016/j.yjmcc.2012.12.016 23291430

[85] Kamota T, Li TS, Morikage N, Murakami M, Ohshima M, Kubo M, Kobayashi T, Mikamo A, Ikeda Y, Matsuzaki M, Hamano K. Ischemic pre-conditioning enhances the mobilization and recruitment of bone marrow stem cells to protect against ischemia/reperfusion injury in the late phase. J Am Coll Cardiol. 2009; 53:1814–22. 10.1016/j.jacc.2009.02.015 19422991

[86] Hepponstall M, Ignjatovic V, Binos S, Monagle P, Jones B, Cheung MH, d’Udekem Y, Konstantinov IE. Remote ischemic preconditioning (RIPC) modifies plasma proteome in humans. PLoS One. 2012; 7:e48284. 10.1371/journal.pone.0048284 23139772 PMC3489679

[87] Zare Mehrjerdi F, Aboutaleb N, Habibey R, Ajami M, Soleimani M, Arabian M, Niknazar S, Hossein Davoodi S, Pazoki-Toroudi H. Increased phosphorylation of mTOR is involved in remote ischemic preconditioning of hippocampus in mice. Brain Res. 2013; 1526:94–101. 10.1016/j.brainres.2013.06.018 23806777

[88] Xie R, Wang P, Ji X, Zhao H. Ischemic post-conditioning facilitates brain recovery after stroke by promoting Akt/mTOR activity in nude rats. J Neurochem. 2013; 127:723–32. 10.1111/jnc.12342 23777415 PMC3875603

[89] Rohailla S, Clarizia N, Sourour M, Sourour W, Gelber N, Wei C, Li J, Redington AN. Acute, delayed and chronic remote ischemic conditioning is associated with downregulation of mTOR and enhanced autophagy signaling. PLoS One. 2014; 9:e111291. 10.1371/journal.pone.0111291 25347774 PMC4210174

[90] Lalu MM, Csonka C, Giricz Z, Csont T, Schulz R, Ferdinandy P. Preconditioning decreases ischemia/reperfusion-induced release and activation of matrix metalloproteinase-2. Biochem Biophys Res Commun. 2002; 296:937–41. 10.1016/S0006-291X(02)02019-3 12200138

[91] Zhang FY, Chen XC, Ren HM, Bao WM. Effects of ischemic preconditioning on blood-brain barrier permeability and MMP-9 expression of ischemic brain. Neurol Res. 2006; 28:21–24. 10.1179/016164106X91825 16464358

[92] Heusch G. Cardioprotection: chances and challenges of its translation to the clinic. Lancet. 2013; 381:166–75. 10.1016/S0140-6736(12)60916-7 23095318

[93] Zitta K, Meybohm P, Bein B, Heinrich C, Renner J, Cremer J, Steinfath M, Scholz J, Albrecht M. Serum from patients undergoing remote ischemic preconditioning protects cultured human intestinal cells from hypoxia-induced damage: involvement of matrixmetalloproteinase-2 and -9. Mol Med. 2012; 18:29–37. 10.2119/molmed.2011.00278 22009279 PMC3269643

[94] Zitta K, Meybohm P, Bein B, Gruenewald M, Lauer F, Steinfath M, Cremer J, Zacharowski K, Albrecht M. Activities of cardiac tissue matrix metalloproteinases 2 and 9 are reduced by remote ischemic preconditioning in cardiosurgical patients with cardiopulmonary bypass. J Transl Med. 2014; 12:94. 10.1186/1479-5876-12-94 24712447 PMC4234318

[95] Pell TJ, Baxter GF, Yellon DM, Drew GM. Renal ischemia preconditions myocardium: role of adenosine receptors and ATP-sensitive potassium channels. Am J Physiol. 1998; 275:H1542–47. 9815059 10.1152/ajpheart.1998.275.5.H1542

[96] Patel HH, Moore J, Hsu AK, Gross GJ. Cardioprotection at a distance: mesenteric artery occlusion protects the myocardium via an opioid sensitive mechanism. J Mol Cell Cardiol. 2002; 34:1317–23. 10.1006/jmcc.2002.2072 12392992

[97] Dong JH, Liu YX, Ji ES, He RR. [Limb ischemic preconditioning reduces infarct size following myocardial ischemia-reperfusion in rats]. Sheng Li Xue Bao. 2004; 56:41–46. Limb ischemic preconditioning reduces infarct size following myocardial ischemia-reperfusion in rats 14985828

[98] Randhawa PK, Jaggi AS. Unraveling the role of adenosine in remote ischemic preconditioning-induced cardioprotection. Life Sci. 2016; 155:140–46. 10.1016/j.lfs.2016.05.009 27157518

[99] Schulte G, Sommerschild H, Yang J, Tokuno S, Goiny M, Lövdahl C, Johansson B, Fredholm BB, Valen G. Adenosine A receptors are necessary for protection of the murine heart by remote, delayed adaptation to ischaemia. Acta Physiol Scand. 2004; 182:133–43. 10.1111/j.1365-201X.2004.01350.x 15450109

[100] Liem DA, Verdouw PD, Ploeg H, Kazim S, Duncker DJ. Sites of action of adenosine in interorgan preconditioning of the heart. Am J Physiol Heart Circ Physiol. 2002; 283:H29–37. 10.1152/ajpheart.01031.2001 12063271

[101] Leung CH, Wang L, Nielsen JM, Tropak MB, Fu YY, Kato H, Callahan J, Redington AN, Caldarone CA. Remote cardioprotection by transfer of coronary effluent from ischemic preconditioned rabbit heart preserves mitochondrial integrity and function via adenosine receptor activation. Cardiovasc Drugs Ther. 2014; 28:7–17. 10.1007/s10557-013-6489-2 24018748

[102] Steensrud T, Li J, Dai X, Manlhiot C, Kharbanda RK, Tropak M, Redington A. Pretreatment with the nitric oxide donor SNAP or nerve transection blocks humoral preconditioning by remote limb ischemia or intra-arterial adenosine. Am J Physiol Heart Circ Physiol. 2010; 299:H1598–603. 10.1152/ajpheart.00396.2010 20802131

[103] Hu S, Dong H, Zhang H, Wang S, Hou L, Chen S, Zhang J, Xiong L. Noninvasive limb remote ischemic preconditioning contributes neuroprotective effects via activation of adenosine A1 receptor and redox status after transient focal cerebral ischemia in rats. Brain Res. 2012; 1459:81–90. 10.1016/j.brainres.2012.04.017 22560096

[104] An MY, Li Y, Chen WH, Zhang Y, Wu YN, Sun K, Pan YY, Yin YQ, Lou JS. Effects of non-invasive remote ischemic conditioning on rehabilitation after myocardial infarction. Biochem Biophys Res Commun. 2017; 488:278–84. 10.1016/j.bbrc.2017.05.014 28479248

[105] Jones WK, Fan GC, Liao S, Zhang JM, Wang Y, Weintraub NL, Kranias EG, Schultz JE, Lorenz J, Ren X. Peripheral nociception associated with surgical incision elicits remote nonischemic cardioprotection via neurogenic activation of protein kinase C signaling. Circulation. 2009 (Suppl ); 120:S1–9. 10.1161/CIRCULATIONAHA.108.843938 19752352 PMC2845316

[106] Pedersen CM, Schmidt MR, Barnes G, Bøtker HE, Kharbanda RK, Newby DE, Cruden NL. Bradykinin does not mediate remote ischaemic preconditioning or ischaemia-reperfusion injury in vivo in man. Heart. 2011; 97:1857–61. 10.1136/heartjnl-2011-300323 21873443

[107] Saxena P, Shaw OM, Misso NL, Naran A, Shehatha J, Newman MA, d’Udekem Y, Thompson PJ, Konstantinov IE. Remote ischemic preconditioning stimulus decreases the expression of kinin receptors in human neutrophils. J Surg Res. 2011; 171:311–16. 10.1016/j.jss.2009.11.011 20189583

[108] Saxena P, Aggarwal S, Misso NL, Passage J, Newman MA, Thompson PJ, d’Udekem Y, Praporski S, Konstantinov IE. Remote ischaemic preconditioning down-regulates kinin receptor expression in neutrophils of patients undergoing heart surgery. Interact Cardiovasc Thorac Surg. 2013; 17:653–58. 10.1093/icvts/ivt279 23814135 PMC3781800

[109] Silachev DN, Isaev NK, Pevzner IB, Zorova LD, Stelmashook EV, Novikova SV, Plotnikov EY, Skulachev VP, Zorov DB. The mitochondria-targeted antioxidants and remote kidney preconditioning ameliorate brain damage through kidney-to-brain cross-talk. PLoS One. 2012; 7:e51553. 10.1371/journal.pone.0051553 23272118 PMC3522699

[110] Hajrasouliha AR, Tavakoli S, Ghasemi M, Jabehdar-Maralani P, Sadeghipour H, Ebrahimi F, Dehpour AR. Endogenous cannabinoids contribute to remote ischemic preconditioning via cannabinoid CB2 receptors in the rat heart. Eur J Pharmacol. 2008; 579:246–52. 10.1016/j.ejphar.2007.09.034 17950273

[111] Ren C, Wang P, Wang B, Li N, Li W, Zhang C, Jin K, Ji X. Limb remote ischemic per-conditioning in combination with post-conditioning reduces brain damage and promotes neuroglobin expression in the rat brain after ischemic stroke. Restor Neurol Neurosci. 2015; 33:369–79. 10.3233/RNN-140413 25868435 PMC4923706

[112] Konstantinov IE, Arab S, Kharbanda RK, Li J, Cheung MM, Cherepanov V, Downey GP, Liu PP, Cukerman E, Coles JG, Redington AN. The remote ischemic preconditioning stimulus modifies inflammatory gene expression in humans. Physiol Genomics. 2004; 19:143–50. 10.1152/physiolgenomics.00046.2004 15304621

[113] Konstantinov IE, Arab S, Li J, Coles JG, Boscarino C, Mori A, Cukerman E, Dawood F, Cheung MM, Shimizu M, Liu PP, Redington AN. The remote ischemic preconditioning stimulus modifies gene expression in mouse myocardium. J Thorac Cardiovasc Surg. 2005; 130:1326–32. 10.1016/j.jtcvs.2005.03.050 16256785

[114] Della-Morte D, Guadagni F, Palmirotta R, Ferroni P, Testa G, Cacciatore F, Abete P, Rengo F, Perez-Pinzon MA, Sacco RL, Rundek T. Genetics and genomics of ischemic tolerance: focus on cardiac and cerebral ischemic preconditioning. Pharmacogenomics. 2012; 13:1741–57. 10.2217/pgs.12.157 23171338

[115] Shimizu M, Saxena P, Konstantinov IE, Cherepanov V, Cheung MM, Wearden P, Zhangdong H, Schmidt M, Downey GP, Redington AN. Remote ischemic preconditioning decreases adhesion and selectively modifies functional responses of human neutrophils. J Surg Res. 2010; 158:155–61. 10.1016/j.jss.2008.08.010 19540519

[116] Ramagiri S, Taliyan R. Protective effect of remote limb post conditioning via upregulation of heme oxygenase-1/BDNF pathway in rat model of cerebral ischemic reperfusion injury. Brain Res. 2017; 1669:44–54. 10.1016/j.brainres.2017.05.016 28535981

[117] Castillo J, Moro MA, Blanco M, Leira R, Serena J, Lizasoain I, Dávalos A. The release of tumor necrosis factor-alpha is associated with ischemic tolerance in human stroke. Ann Neurol. 2003; 54:811–19. 10.1002/ana.10765 14681891

[118] Kobayashi SD, DeLeo FR. Role of neutrophils in innate immunity: a systems biology-level approach. Wiley Interdiscip Rev Syst Biol Med. 2009; 1:309–33. 10.1002/wsbm.32 20836000 PMC3501127

[119] Smith RM, Suleman N, McCarthy J, Sack MN. Classic ischemic but not pharmacologic preconditioning is abrogated following genetic ablation of the TNFalpha gene. Cardiovasc Res. 2002; 55:553–60. 10.1016/S0008-6363(02)00283-3 12160952

[120] Teoh N, Field J, Farrell G. Interleukin-6 is a key mediator of the hepatoprotective and pro-proliferative effects of ischaemic preconditioning in mice. J Hepatol. 2006; 45:20–27. 10.1016/j.jhep.2006.01.039 16600417

[121] Zuurbier CJ, Jong WM, Eerbeek O, Koeman A, Pulskens WP, Butter LM, Leemans JC, Hollmann MW. Deletion of the innate immune NLRP3 receptor abolishes cardiac ischemic preconditioning and is associated with decreased Il-6/STAT3 signaling. PLoS One. 2012; 7:e40643. 10.1371/journal.pone.0040643 22848390 PMC3407219

[122] Cai ZP, Parajuli N, Zheng X, Becker L. Remote ischemic preconditioning confers late protection against myocardial ischemia-reperfusion injury in mice by upregulating interleukin-10. Basic Res Cardiol. 2012; 107:277. 10.1007/s00395-012-0277-1 22752341 PMC3596418

[123] Liu ZJ, Chen C, Li XR, Ran YY, Xu T, Zhang Y, Geng XK, Zhang Y, Du HS, Leak RK, Ji XM, Hu XM. Remote ischemic preconditioning-mediated neuroprotection against stroke is associated with significant alterations in peripheral immune responses. CNS Neurosci Ther. 2016; 22:43–52. 10.1111/cns.12448 26384716 PMC6492849

[124] Chen C, Jiang W, Liu Z, Li F, Yang J, Zhao Y, Ran Y, Meng Y, Ji X, Geng X, Du H, Hu X. Splenic responses play an important role in remote ischemic preconditioning-mediated neuroprotection against stroke. J Neuroinflammation. 2018; 15:167. 10.1186/s12974-018-1190-9 29807548 PMC5972448

[125] Wei M, Xin P, Li S, Tao J, Li Y, Li J, Liu M, Li J, Zhu W, Redington AN. Repeated remote ischemic postconditioning protects against adverse left ventricular remodeling and improves survival in a rat model of myocardial infarction. Circ Res. 2011; 108:1220–25. 10.1161/CIRCRESAHA.110.236190 21474817

[126] Costa FL, Yamaki VN, Gonçalves TB, Coelho JV, Percário S, Brito MV. Combined remote ischemic perconditioning and local postconditioning on liver ischemia-reperfusion injury. J Surg Res. 2014; 192:98–102. 10.1016/j.jss.2014.05.046 24952413

[127] Zhou M, Xia ZY, Lei SQ, Leng Y, Xue R. Role of mitophagy regulated by Parkin/DJ-1 in remote ischemic postconditioning-induced mitigation of focal cerebral ischemia-reperfusion. Eur Rev Med Pharmacol Sci. 2015; 19:4866–71. 26744879

[128] Oliveira RC, Brito MV, Ribeiro RF, Oliveira LO, Monteiro AM, Brandão FM, Cavalcante LC, Gouveia EH, Henriques HY. Influence of remote ischemic conditioning and tramadol hydrochloride on oxidative stress in kidney ischemia/reperfusion injury in rats. Acta Cir Bras. 2017; 32:229–35. 10.1590/s0102-865020170030000007 28403347

[129] Lotfollahi H, Mohammadi M, Ghaffari S, Badalzadeh R, Sohrabi B, Aslanabadi N, Separham A, Golmohammadi A, Abbasnejad A, Roshani M. Effect of remote ischemic post-conditioning on oxidative stress in blood of STEMI patients treated with primary angioplasty. J Cardiovasc Thorac Res. 2016; 8:113–18. 10.15171/jcvtr.2016.24 27777696 PMC5075359

[130] Qi ZF, Luo YM, Liu XR, Wang RL, Zhao HP, Yan F, Song ZJ, Luo M, Ji XM. AKT/GSK3β-dependent autophagy contributes to the neuroprotection of limb remote ischemic postconditioning in the transient cerebral ischemic rat model. CNS Neurosci Ther. 2012; 18:965–73. 10.1111/cns.12016 23191937 PMC6493377

[131] Qi Z, Dong W, Shi W, Wang R, Zhang C, Zhao Y, Ji X, Liu KJ, Luo Y. Bcl-2 phosphorylation triggers autophagy switch and reduces mitochondrial damage in limb remote ischemic conditioned rats after ischemic stroke. Transl Stroke Res. 2015; 6:198–206. 10.1007/s12975-015-0393-y 25744447

[132] Guo H, Zhao L, Wang B, Li X, Bai H, Liu H, Yue L, Guo W, Bian Z, Gao L, Feng D, Qu Y. Remote limb ischemic postconditioning protects against cerebral ischemia-reperfusion injury by activating AMPK-dependent autophagy. Brain Res Bull. 2018; 139:105–13. 10.1016/j.brainresbull.2018.02.013 29452253

[133] Chen GZ, Shan XY, Li XS, Tao HM. Remote ischemic postconditioning protects the brain from focal ischemia/reperfusion injury by inhibiting autophagy through the mTOR/p70S6K pathway. Neurol Res. 2018; 40:182–88. 10.1080/01616412.2018.1424696 29369005

[134] Gao L, Jiang T, Guo J, Liu Y, Cui G, Gu L, Su L, Zhang Y. Inhibition of autophagy contributes to ischemic postconditioning-induced neuroprotection against focal cerebral ischemia in rats. PLoS One. 2012; 7:e46092. 10.1371/journal.pone.0046092 23029398 PMC3461004

[135] Wang J, Han D, Sun M, Feng J. A Combination of Remote Ischemic Perconditioning and Cerebral Ischemic Postconditioning Inhibits Autophagy to Attenuate Plasma HMGB1 and Induce Neuroprotection Against Stroke in Rat. J Mol Neurosci. 2016; 58:424–31. 10.1007/s12031-016-0724-9 26852332

[136] Halcox JP, Schenke WH, Zalos G, Mincemoyer R, Prasad A, Waclawiw MA, Nour KR, Quyyumi AA. Prognostic value of coronary vascular endothelial dysfunction. Circulation. 2002; 106:653–58. 10.1161/01.CIR.0000025404.78001.D8 12163423

[137] Higashi Y. Assessment of endothelial function. History, methodological aspects, and clinical perspectives. Int Heart J. 2015; 56:125–34. 10.1536/ihj.14-385 25740586

[138] Jones H, Hopkins N, Bailey TG, Green DJ, Cable NT, Thijssen DH. Seven-day remote ischemic preconditioning improves local and systemic endothelial function and microcirculation in healthy humans. Am J Hypertens. 2014; 27:918–25. 10.1093/ajh/hpu004 24627443

[139] Jones H, Nyakayiru J, Bailey TG, Green DJ, Cable NT, Sprung VS, Hopkins ND, Thijssen DH. Impact of eight weeks of repeated ischaemic preconditioning on brachial artery and cutaneous microcirculatory function in healthy males. Eur J Prev Cardiol. 2015; 22:1083–87. 10.1177/2047487314547657 25147345

[140] Liang Y, Li YP, He F, Liu XQ, Zhang JY. Long-term, regular remote ischemic preconditioning improves endothelial function in patients with coronary heart disease. Braz J Med Biol Res. 2015; 48:568–76. 10.1590/1414-431X20144452 25923462 PMC4470317

[141] Corcoran D, Young R, Cialdella P, McCartney P, Bajrangee A, Hennigan B, Collison D, Carrick D, Shaukat A, Good R, Watkins S, McEntegart M, Watt J, et al. The effects of remote ischaemic preconditioning on coronary artery function in patients with stable coronary artery disease. Int J Cardiol. 2018; 252:24–30. 10.1016/j.ijcard.2017.10.082 29249435 PMC5761717

[142] Meng R, Ding Y, Asmaro K, Brogan D, Meng L, Sui M, Shi J, Duan Y, Sun Z, Yu Y, Jia J, Ji X. Ischemic conditioning is safe and effective for octo- and nonagenarians in stroke prevention and treatment. Neurotherapeutics. 2015; 12:667–77. 10.1007/s13311-015-0358-6 25956401 PMC4489956

[143] Meng R, Asmaro K, Meng L, Liu Y, Ma C, Xi C, Li G, Ren C, Luo Y, Ling F, Jia J, Hua Y, Wang X, et al. Upper limb ischemic preconditioning prevents recurrent stroke in intracranial arterial stenosis. Neurology. 2012; 79:1853–61. 10.1212/WNL.0b013e318271f76a 23035060

[144] Khan MB, Hafez S, Hoda MN, Baban B, Wagner J, Awad ME, Sangabathula H, Haigh S, Elsalanty M, Waller JL, Hess DC. Chronic remote ischemic conditioning is cerebroprotective and induces vascular remodeling in a VCID model. Transl Stroke Res. 2018; 9:51–63. 10.1007/s12975-017-0555-1 28755277 PMC5750336

[145] Khan MB, Hoda MN, Vaibhav K, Giri S, Wang P, Waller JL, Ergul A, Dhandapani KM, Fagan SC, Hess DC. Remote ischemic postconditioning: harnessing endogenous protection in a murine model of vascular cognitive impairment. Transl Stroke Res. 2015; 6:69–77. 10.1007/s12975-014-0374-6 25351177 PMC4297613

[146] Hougaard KD, Hjort N, Zeidler D, Sørensen L, Nørgaard A, Hansen TM, von Weitzel-Mudersbach P, Simonsen CZ, Damgaard D, Gottrup H, Svendsen K, Rasmussen PV, Ribe LR, et al. Remote ischemic perconditioning as an adjunct therapy to thrombolysis in patients with acute ischemic stroke: a randomized trial. Stroke. 2014; 45:159–67. 10.1161/STROKEAHA.113.001346 24203849

[147] England TJ, Hedstrom A, O’Sullivan S, Donnelly R, Barrett DA, Sarmad S, Sprigg N, Bath PM. RECAST (Remote Ischemic Conditioning After Stroke Trial): A Pilot Randomized Placebo Controlled Phase II Trial in Acute Ischemic Stroke. Stroke. 2017; 48:1412–15. 10.1161/STROKEAHA.116.016429 28265014

[148] Wei M, Huo K, Liu R, Yang J, Cheng Y, Chang S, Ren D, Luo G. The Design and Rationale of a Clinical Trial Evaluating Limb Postconditioning in Young Patients with Intracranial Arterial Stenosis. J Stroke Cerebrovasc Dis. 2016; 25:2506–12. 10.1016/j.jstrokecerebrovasdis.2016.06.027 27431451

[149] Hou C, Duan J, Luo Y, Meng R, Li S, Yao C, Ding Y, Zhang H, Wang Y, Zhao G, Zhang J, Ji X. Remote limb ischemic conditioning treatment for intracranial atherosclerotic stenosis patients. Int J Stroke. 2016; 11:831–38. 10.1177/1747493016654489 27312678

[150] Koch S, Katsnelson M, Dong C, Perez-Pinzon M. Remote ischemic limb preconditioning after subarachnoid hemorrhage: a phase Ib study of safety and feasibility. Stroke. 2011; 42:1387–91. 10.1161/STROKEAHA.110.605840 21415404 PMC3082628

[151] Bilgin-Freiert A, Dusick JR, Stein NR, Etchepare M, Vespa P, Gonzalez NR. Muscle microdialysis to confirm sublethal ischemia in the induction of remote ischemic preconditioning. Transl Stroke Res. 2012; 3:266–72. 10.1007/s12975-012-0153-1 24323782

[152] Gonzalez NR, Hamilton R, Bilgin-Freiert A, Dusick J, Vespa P, Hu X, Asgari S. Cerebral hemodynamic and metabolic effects of remote ischemic preconditioning in patients with subarachnoid hemorrhage. Acta Neurochir Suppl (Wien). 2013; 115:193–98. 22890668 10.1007/978-3-7091-1192-5_36

[153] Gonzalez NR, Connolly M, Dusick JR, Bhakta H, Vespa P. Phase I clinical trial for the feasibility and safety of remote ischemic conditioning for aneurysmal subarachnoid hemorrhage. Neurosurgery. 2014; 75:590–98. 10.1227/NEU.0000000000000514 25072112 PMC4205274

[154] Nikkola E, Laiwalla A, Ko A, Alvarez M, Connolly M, Ooi YC, Hsu W, Bui A, Pajukanta P, Gonzalez NR. Remote ischemic conditioning alters methylation and expression of cell cycle genes in aneurysmal subarachnoid hemorrhage. Stroke. 2015; 46:2445–51. 10.1161/STROKEAHA.115.009618 26251247 PMC4550559

[155] Laiwalla AN, Ooi YC, Liou R, Gonzalez NR. Matched cohort analysis of the effects of limb remote ischemic conditioning in patients with aneurysmal subarachnoid hemorrhage. Transl Stroke Res. 2016; 7:42–48. 10.1007/s12975-015-0437-3 26630942 PMC4724226

[156] Walsh SR, Nouraei SA, Tang TY, Sadat U, Carpenter RH, Gaunt ME. Remote ischemic preconditioning for cerebral and cardiac protection during carotid endarterectomy: results from a pilot randomized clinical trial. Vasc Endovascular Surg. 2010; 44:434–39. 10.1177/1538574410369709 20484064

[157] Zhao W, Meng R, Ma C, Hou B, Jiao L, Zhu F, Wu W, Shi J, Duan Y, Zhang R, Zhang J, Sun Y, Zhang H, et al. Safety and efficacy of remote ischemic preconditioning in patients with severe carotid artery stenosis before carotid artery stenting: a proof-of-concept, randomized controlled trial. Circulation. 2017; 135:1325–35. 10.1161/CIRCULATIONAHA.116.024807 28174194 PMC5802341

[158] Mi T, Yu F, Ji X, Sun Y, Qu D. The Interventional Effect of Remote Ischemic Preconditioning on Cerebral Small Vessel Disease: A Pilot Randomized Clinical Trial. Eur Neurol. 2016; 76:28–34. 10.1159/000447536 27351719

[159] US National Library of Medicine. ClinicalTrials.gov

[160] Wang Y, Meng R, Song H, Liu G, Hua Y, Cui D, Zheng L, Feng W, Liebeskind DS, Fisher M, Ji X. Remote Ischemic Conditioning May Improve Outcomes of Patients With Cerebral Small-Vessel Disease. Stroke. 2017; 48:3064–72. 10.1161/STROKEAHA.117.017691 29042490

[161] Joseph B, Pandit V, Zangbar B, Kulvatunyou N, Khalil M, Tang A, O’Keeffe T, Gries L, Vercruysse G, Friese RS, Rhee P. Secondary brain injury in trauma patients: the effects of remote ischemic conditioning. J Trauma Acute Care Surg. 2015; 78:698–703. 10.1097/TA.0000000000000584 25742251

[162] Günaydin B, Cakici I, Soncul H, Kalaycioglu S, Cevik C, Sancak B, Kanzik I, Karadenizli Y. Does remote organ ischaemia trigger cardiac preconditioning during coronary artery surgery? Pharmacol Res. 2000; 41:493–96. 10.1006/phrs.1999.0611 10704275

[163] Hausenloy DJ, Mwamure PK, Venugopal V, Harris J, Barnard M, Grundy E, Ashley E, Vichare S, Di Salvo C, Kolvekar S, Hayward M, Keogh B, MacAllister RJ, Yellon DM. Effect of remote ischaemic preconditioning on myocardial injury in patients undergoing coronary artery bypass graft surgery: a randomised controlled trial. Lancet. 2007; 370:575–79. 10.1016/S0140-6736(07)61296-3 17707752

[164] Venugopal V, Hausenloy DJ, Ludman A, Di Salvo C, Kolvekar S, Yap J, Lawrence D, Bognolo J, Yellon DM. Remote ischaemic preconditioning reduces myocardial injury in patients undergoing cardiac surgery with cold-blood cardioplegia: a randomised controlled trial. Heart. 2009; 95:1567–71. 10.1136/hrt.2008.155770 19508973

[165] Thielmann M, Kottenberg E, Boengler K, Raffelsieper C, Neuhaeuser M, Peters J, Jakob H, Heusch G. Remote ischemic preconditioning reduces myocardial injury after coronary artery bypass surgery with crystalloid cardioplegic arrest. Basic Res Cardiol. 2010; 105:657–64. 10.1007/s00395-010-0104-5 20495811

[166] Wagner R, Piler P, Bedanova H, Adamek P, Grodecka L, Freiberger T. Myocardial injury is decreased by late remote ischaemic preconditioning and aggravated by tramadol in patients undergoing cardiac surgery: a randomised controlled trial. Interact Cardiovasc Thorac Surg. 2010; 11:758–62. 10.1510/icvts.2010.243600 20847065

[167] Kottenberg E, Thielmann M, Bergmann L, Heine T, Jakob H, Heusch G, Peters J. Protection by remote ischemic preconditioning during coronary artery bypass graft surgery with isoflurane but not propofol - a clinical trial. Acta Anaesthesiol Scand. 2012; 56:30–38. 10.1111/j.1399-6576.2011.02585.x 22103808

[168] Ali N, Rizwi F, Iqbal A, Rashid A. Induced remote ischemic pre-conditioning on ischemia-reperfusion injury in patients undergoing coronary artery bypass. J Coll Physicians Surg Pak. 2010; 20:427–31. 20642939

[169] Thielmann M, Kottenberg E, Kleinbongard P, Wendt D, Gedik N, Pasa S, Price V, Tsagakis K, Neuhäuser M, Peters J, Jakob H, Heusch G. Cardioprotective and prognostic effects of remote ischaemic preconditioning in patients undergoing coronary artery bypass surgery: a single-centre randomised, double-blind, controlled trial. Lancet. 2013; 382:597–604. 10.1016/S0140-6736(13)61450-6 23953384

[170] Candilio L, Malik A, Ariti C, Barnard M, Di Salvo C, Lawrence D, Hayward M, Yap J, Roberts N, Sheikh A, Kolvekar S, Hausenloy DJ, Yellon DM. Effect of remote ischaemic preconditioning on clinical outcomes in patients undergoing cardiac bypass surgery: a randomised controlled clinical trial. Heart. 2015; 101:185–92. 10.1136/heartjnl-2014-306178 25252696

[171] Hausenloy DJ, Candilio L, Evans R, Ariti C, Jenkins DP, Kolvekar S, Knight R, Kunst G, Laing C, Nicholas J, Pepper J, Robertson S, Xenou M, et al, and ERICCA Trial Investigators. Remote ischemic preconditioning and outcomes of cardiac surgery. N Engl J Med. 2015; 373:1408–17. 10.1056/NEJMoa1413534 26436207

[172] Meybohm P, Bein B, Brosteanu O, Cremer J, Gruenewald M, Stoppe C, Coburn M, Schaelte G, Böning A, Niemann B, Roesner J, Kletzin F, Strouhal U, et al, and RIPHeart Study Collaborators. A multicenter trial of remote ischemic preconditioning for heart surgery. N Engl J Med. 2015; 373:1397–407. 10.1056/NEJMoa1413579 26436208

[173] Rahman IA, Mascaro JG, Steeds RP, Frenneaux MP, Nightingale P, Gosling P, Townsend P, Townend JN, Green D, Bonser RS. Remote ischemic preconditioning in human coronary artery bypass surgery: from promise to disappointment? Circulation. 2010 (Suppl ); 122:S53–59. 10.1161/CIRCULATIONAHA.109.926667 20837926

[174] Lucchinetti E, Bestmann L, Feng J, Freidank H, Clanachan AS, Finegan BA, Zaugg M. Remote ischemic preconditioning applied during isoflurane inhalation provides no benefit to the myocardium of patients undergoing on-pump coronary artery bypass graft surgery: lack of synergy or evidence of antagonism in cardioprotection? Anesthesiology. 2012; 116:296–310. 10.1097/ALN.0b013e318242349a 22222469

[175] Young PJ, Dalley P, Garden A, Horrocks C, La Flamme A, Mahon B, Miller J, Pilcher J, Weatherall M, Williams J, Young W, Beasley R. A pilot study investigating the effects of remote ischemic preconditioning in high-risk cardiac surgery using a randomised controlled double-blind protocol. Basic Res Cardiol. 2012; 107:256. 10.1007/s00395-012-0256-6 22406977

[176] Iliodromitis EK, Kyrzopoulos S, Paraskevaidis IA, Kolocassides KG, Adamopoulos S, Karavolias G, Kremastinos DT. Increased C reactive protein and cardiac enzyme levels after coronary stent implantation. Is there protection by remote ischaemic preconditioning? Heart. 2006; 92:1821–26. 10.1136/hrt.2006.089060 16855045 PMC1861265

[177] Hoole SP, Heck PM, Sharples L, Khan SN, Duehmke R, Densem CG, Clarke SC, Shapiro LM, Schofield PM, O’Sullivan M, Dutka DP. Cardiac Remote Ischemic Preconditioning in Coronary Stenting (CRISP Stent) Study: a prospective, randomized control trial. Circulation. 2009; 119:820–27. 10.1161/CIRCULATIONAHA.108.809723 19188504

[178] Hoole SP, Khan SN, White PA, Heck PM, Kharbanda RK, Densem CG, Clarke SC, Shapiro LM, Schofield PM, O’Sullivan M, Dutka DP. Remote ischaemic pre-conditioning does not attenuate ischaemic left ventricular dysfunction in humans. Eur J Heart Fail. 2009; 11:497–505. 10.1093/eurjhf/hfp040 19386814

[179] Ahmed RM, Mohamed HA, Ashraf M, Maithili S, Nabil F, Rami R, Mohamed TI. Effect of remote ischemic preconditioning on serum troponin T level following elective percutaneous coronary intervention. Catheter Cardiovasc Interv. 2013; 82:E647–53. 10.1002/ccd.24825 23404916

[180] Luo SJ, Zhou YJ, Shi DM, Ge HL, Wang JL, Liu RF. Remote ischemic preconditioning reduces myocardial injury in patients undergoing coronary stent implantation. Can J Cardiol. 2013; 29:1084–89. 10.1016/j.cjca.2012.11.022 23414904

[181] Zografos TA, Katritsis GD, Tsiafoutis I, Bourboulis N, Katsivas A, Katritsis DG. Effect of one-cycle remote ischemic preconditioning to reduce myocardial injury during percutaneous coronary intervention. Am J Cardiol. 2014; 113:2013–17. 10.1016/j.amjcard.2014.03.043 24793669

[182] Liu Z, Wang YL, Hua Q, Chu YY, Ji XM. Late remote ischemic preconditioning provides benefit to patients undergoing elective percutaneous coronary intervention. Cell Biochem Biophys. 2014; 70:437–42. 10.1007/s12013-014-9936-1 25015066

[183] Davies WR, Brown AJ, Watson W, McCormick LM, West NE, Dutka DP, Hoole SP. Remote ischemic preconditioning improves outcome at 6 years after elective percutaneous coronary intervention: the CRISP stent trial long-term follow-up. Circ Cardiovasc Interv. 2013; 6:246–51. 10.1161/CIRCINTERVENTIONS.112.000184 23696599

[184] Prasad A, Gössl M, Hoyt J, Lennon RJ, Polk L, Simari R, Holmes DR Jr, Rihal CS, Lerman A. Remote ischemic preconditioning immediately before percutaneous coronary intervention does not impact myocardial necrosis, inflammatory response, and circulating endothelial progenitor cell counts: a single center randomized sham controlled trial. Catheter Cardiovasc Interv. 2013; 81:930–36. 10.1002/ccd.24443 22517646

[185] Xu X, Zhou Y, Luo S, Zhang W, Zhao Y, Yu M, Ma Q, Gao F, Shen H, Zhang J. Effect of remote ischemic preconditioning in the elderly patients with coronary artery disease with diabetes mellitus undergoing elective drug-eluting stent implantation. Angiology. 2014; 65:660–66. 10.1177/0003319713507332 24163121

[186] Lavi S, D’Alfonso S, Diamantouros P, Camuglia A, Garg P, Teefy P, Jablonsky G, Sridhar K, Lavi R. Remote ischemic postconditioning during percutaneous coronary interventions: remote ischemic postconditioning-percutaneous coronary intervention randomized trial. Circ Cardiovasc Interv. 2014; 7:225–32. 10.1161/CIRCINTERVENTIONS.113.000948 24692535

[187] Pei H, Wu Y, Wei Y, Yang Y, Teng S, Zhang H. Remote ischemic preconditioning reduces perioperative cardiac and renal events in patients undergoing elective coronary intervention: a meta-analysis of 11 randomized trials. PLoS One. 2014; 9:e115500. 10.1371/journal.pone.0115500 25551671 PMC4281209

[188] Zografos TA, Katritsis GD, Katritsis DG. Remote ischemic preconditioning reduces peri-procedural myocardial injury in elective percutaneous coronary intervention: a meta-analysis. Int J Cardiol. 2014; 173:530–32. 10.1016/j.ijcard.2014.03.026 24681008

[189] Yu Q, Zhou L, Liu L, Cong L, Wang Y, Ge T, Lin D. Stromal cell-derived factor-1 alpha alleviates hypoxic-ischemic brain damage in mice. Biochem Biophys Res Commun. 2015; 464:447–52. 10.1016/j.bbrc.2015.06.135 26145601

[190] Le Page S, Bejan-Angoulvant T, Angoulvant D, Prunier F. Remote ischemic conditioning and cardioprotection: a systematic review and meta-analysis of randomized clinical trials. Basic Res Cardiol. 2015; 110:11. 10.1007/s00395-015-0467-8 25653117

[191] Moretti C, Cavallero E, D’Ascenzo F, Cerrato E, Zoccai GB, Omedè P, Presutti DG, Lefevre T, Sanguineti F, Picchi A, Palazzuoli A, Carini G, Giammaria M, et al. The EUROpean and Chinese cardiac and renal Remote Ischemic Preconditioning Study (EURO-CRIPS): study design and methods. J Cardiovasc Med (Hagerstown). 2015; 16:246–52. 10.2459/JCM.0000000000000098 24859616

[192] Munk K, Andersen NH, Schmidt MR, Nielsen SS, Terkelsen CJ, Sloth E, Bøtker HE, Nielsen TT, Poulsen SH. Remote ischemic conditioning in patients with myocardial infarction treated with primary angioplasty: impact on left ventricular function assessed by comprehensive echocardiography and gated single-photon emission CT. Circ Cardiovasc Imaging. 2010; 3:656–62. 10.1161/CIRCIMAGING.110.957340 20826592

[193] Sloth AD, Schmidt MR, Munk K, Kharbanda RK, Redington AN, Schmidt M, Pedersen L, Sørensen HT, Bøtker HE, and CONDI Investigators. Improved long-term clinical outcomes in patients with ST-elevation myocardial infarction undergoing remote ischaemic conditioning as an adjunct to primary percutaneous coronary intervention. Eur Heart J. 2014; 35:168–75. 10.1093/eurheartj/eht369 24031025

[194] Crimi G, Pica S, Raineri C, Bramucci E, De Ferrari GM, Klersy C, Ferlini M, Marinoni B, Repetto A, Romeo M, Rosti V, Massa M, Raisaro A, et al. Remote ischemic post-conditioning of the lower limb during primary percutaneous coronary intervention safely reduces enzymatic infarct size in anterior myocardial infarction: a randomized controlled trial. JACC Cardiovasc Interv. 2013; 6:1055–63. 10.1016/j.jcin.2013.05.011 24156966

[195] White SK, Frohlich GM, Sado DM, Maestrini V, Fontana M, Treibel TA, Tehrani S, Flett AS, Meier P, Ariti C, Davies JR, Moon JC, Yellon DM, Hausenloy DJ. Remote ischemic conditioning reduces myocardial infarct size and edema in patients with ST-segment elevation myocardial infarction. JACC Cardiovasc Interv. 2015 (1 Pt B); 8:178–88. 10.1016/j.jcin.2014.05.015 25240548

[196] Eitel I, Stiermaier T, Rommel KP, Fuernau G, Sandri M, Mangner N, Linke A, Erbs S, Lurz P, Boudriot E, Mende M, Desch S, Schuler G, Thiele H. Cardioprotection by combined intrahospital remote ischaemic perconditioning and postconditioning in ST-elevation myocardial infarction: the randomized LIPSIA CONDITIONING trial. Eur Heart J. 2015; 36:3049–57. 10.1093/eurheartj/ehv463 26385956

[197] Hausenloy DJ, Kharbanda R, Rahbek Schmidt M, Møller UK, Ravkilde J, Okkels Jensen L, Engstrøm T, Garcia Ruiz JM, Radovanovic N, Christensen EF, Sørensen HT, Ramlall M, Bulluck H, et al. Effect of remote ischaemic conditioning on clinical outcomes in patients presenting with an ST-segment elevation myocardial infarction undergoing primary percutaneous coronary intervention. Eur Heart J. 2015; 36:1846–48. 26460398

[198] Gaspar A, Lourenço AP, Pereira MÁ, Azevedo P, Roncon-Albuquerque R Jr, Marques J, Leite-Moreira AF. Randomized controlled trial of remote ischaemic conditioning in ST-elevation myocardial infarction as adjuvant to primary angioplasty (RIC-STEMI). Basic Res Cardiol. 2018; 113:14. 10.1007/s00395-018-0672-3 29516192

[199] Kono Y, Fukuda S, Hanatani A, Nakanishi K, Otsuka K, Taguchi H, Shimada K. Remote ischemic conditioning improves coronary microcirculation in healthy subjects and patients with heart failure. Drug Des Devel Ther. 2014; 8:1175–81. 25210440 10.2147/DDDT.S68715 PMC4154883

[200] Pryds K, Nielsen RR, Jorsal A, Hansen MS, Ringgaard S, Refsgaard J, Kim WY, Petersen AK, Bøtker HE, Schmidt MR. Effect of long-term remote ischemic conditioning in patients with chronic ischemic heart failure. Basic Res Cardiol. 2017; 112:67. 10.1007/s00395-017-0658-6 29071437

[201] Chen L, Zhou Q, Jin H, Zhu K, Zhi H, Chen Z, Ma G. Effects of remote ischaemic conditioning on heart rate variability and cardiac function in patients with mild ischaemic heart failure. Heart Lung Circ. 2018; 27:477–83. 10.1016/j.hlc.2017.03.164 28533100

[202] Venugopal V, Laing CM, Ludman A, Yellon DM, Hausenloy D. Effect of remote ischemic preconditioning on acute kidney injury in nondiabetic patients undergoing coronary artery bypass graft surgery: a secondary analysis of 2 small randomized trials. Am J Kidney Dis. 2010; 56:1043–49. 10.1053/j.ajkd.2010.07.014 20974511 PMC2991586

[203] Zimmerman RF, Ezeanuna PU, Kane JC, Cleland CD, Kempananjappa TJ, Lucas FL, Kramer RS. Ischemic preconditioning at a remote site prevents acute kidney injury in patients following cardiac surgery. Kidney Int. 2011; 80:861–67. 10.1038/ki.2011.156 21677633

[204] Er F, Nia AM, Dopp H, Hellmich M, Dahlem KM, Caglayan E, Kubacki T, Benzing T, Erdmann E, Burst V, Gassanov N. Ischemic preconditioning for prevention of contrast medium-induced nephropathy: randomized pilot RenPro Trial (Renal Protection Trial). Circulation. 2012; 126:296–303. 10.1161/CIRCULATIONAHA.112.096370 22735306

[205] Igarashi G, Iino K, Watanabe H, Ito H. Remote ischemic pre-conditioning alleviates contrast-induced acute kidney injury in patients with moderate chronic kidney disease. Circ J. 2013; 77:3037–44. 10.1253/circj.CJ-13-0171 23986081

[206] Gallagher SM, Jones DA, Kapur A, Wragg A, Harwood SM, Mathur R, Archbold RA, Uppal R, Yaqoob MM. Remote ischemic preconditioning has a neutral effect on the incidence of kidney injury after coronary artery bypass graft surgery. Kidney Int. 2015; 87:473–81. 10.1038/ki.2014.259 25075773

[207] Zarbock A, Schmidt C, Van Aken H, Wempe C, Martens S, Zahn PK, Wolf B, Goebel U, Schwer CI, Rosenberger P, Haeberle H, Görlich D, Kellum JA, Meersch M, and RenalRIPC Investigators. Effect of remote ischemic preconditioning on kidney injury among high-risk patients undergoing cardiac surgery: a randomized clinical trial. JAMA. 2015; 313:2133–41. 10.1001/jama.2015.4189 26024502

[208] Zuo B, Wang F, Song Z, Xu M, Wang G. Using remote ischemic conditioning to reduce acute kidney injury in patients undergoing percutaneous coronary intervention: a meta-analysis. Curr Med Res Opin. 2015; 31:1677–85. 10.1185/03007995.2015.1066766 26154745

[209] Menting TP, Sterenborg TB, de Waal Y, Donders R, Wever KE, Lemson MS, van der Vliet JA, Wetzels JF, SchultzeKool LJ, Warlé MC. Remote Ischemic Preconditioning To Reduce Contrast-Induced Nephropathy: A Randomized Controlled Trial. Eur J Vasc Endovasc Surg. 2015; 50:527–32. 10.1016/j.ejvs.2015.04.002 26015372

[210] Balbir Singh G, Ann SH, Park J, Chung HC, Lee JS, Kim ES, Choi JI, Lee J, Kim SJ, Shin ES. Remote ischemic preconditioning for the prevention of contrast-induced acute kidney injury in diabetics receiving elective percutaneous coronary intervention. PLoS One. 2016; 11:e0164256. 10.1371/journal.pone.0164256 27723839 PMC5056748

[211] Zhou C, Jeon Y, Meybohm P, Zarbock A, Young PJ, Li L, Hausenloy DJ. Renoprotection by remote ischemic conditioning during elective coronary revascularization: A systematic review and meta-analysis of randomized controlled trials. Int J Cardiol. 2016; 222:295–302. 10.1016/j.ijcard.2016.07.176 27498373

[212] Zarbock A, Kellum JA, Van Aken H, Schmidt C, Küllmar M, Rosenberger P, Martens S, Görlich D, Meersch M. Long-term effects of remote ischemic preconditioning on kidney function in high-risk cardiac surgery patients: follow-up results from the RenalRIP Trial. Anesthesiology. 2017; 126:787–98. 10.1097/ALN.0000000000001598 28288051

[213] Chen Y, Zheng H, Wang X, Zhou Z, Luo A, Tian Y. Remote ischemic preconditioning fails to improve early renal function of patients undergoing living-donor renal transplantation: a randomized controlled trial. Transplantation. 2013; 95:e4–6. 10.1097/TP.0b013e3182782f3a 23325011

[214] van den Akker EK, Hesselink DA, Manintveld OC, Lafranca JA, de Bruin RW, Weimar W, Ijzermans JN, Dor FJ. Ischemic postconditioning in human DCD kidney transplantation is feasible and appears safe. Transpl Int. 2014; 27:226–34. 10.1111/tri.12242 24236960

[215] Wu J, Feng X, Huang H, Shou Z, Zhang X, Wang R, Chen Y, Chen J. Remote ischemic conditioning enhanced the early recovery of renal function in recipients after kidney transplantation: a randomized controlled trial. J Surg Res. 2014; 188:303–08. 10.1016/j.jss.2013.06.058 24556231

[216] MacAllister R, Clayton T, Knight R, Robertson S, Nicholas J, Motwani M, Veighey K. (2015). Efficacy and Mechanism Evaluation. REmote preconditioning for Protection Against Ischaemia-Reperfusion in renal transplantation (REPAIR): a multicentre, multinational, double-blind, factorial designed randomised controlled trial. (Southampton (UK): NIHR Journals Library Copyright (c) Queen's Printer and Controller of HMSO 2015. This work was produced by MacAllister et al. under the terms of a commissioning contract issued by the Secretary of State for Health. This issue may be freely reproduced for the purposes of private research and study and extracts (or indeed, the full report) may be included in professional journals provided that suitable acknowledgement is made and the reproduction is not associated with any form of advertising. Applications for commercial reproduction should be addressed to: NIHR Journals Library, National Institute for Health Research, Evaluation, Trials and Studies Coordinating Centre, Alpha House, University of Southampton Science Park, Southampton SO16 7NS, UK.).

[217] Nicholson ML, Pattenden CJ, Barlow AD, Hunter JP, Lee G, Hosgood SA. A double blind randomized clinical trial of remote ischemic conditioning in live donor renal transplantation. Medicine (Baltimore). 2015; 94:e1316. 10.1097/MD.0000000000001316 26252316 PMC4616604

[218] Krogstrup NV, Oltean M, Nieuwenhuijs-Moeke GJ, Dor FJ, Møldrup U, Krag SP, Bibby BM, Birn H, Jespersen B. Remote ischemic conditioning on recipients of deceased renal transplants does not improve early graft function: a multicenter randomized, controlled clinical trial. Am J Transplant. 2017; 17:1042–49. 10.1111/ajt.14075 27696662

[219] Park J, Ann SH, Chung HC, Lee JS, Kim SJ, Garg S, Shin ES. Remote ischemic preconditioning in hemodialysis: a pilot study. Heart Vessels. 2014; 29:58–64. 10.1007/s00380-013-0329-y 23532306

[220] Kim JC, Shim JK, Lee S, Yoo YC, Yang SY, Kwak YL. Effect of combined remote ischemic preconditioning and postconditioning on pulmonary function in valvular heart surgery. Chest. 2012; 142:467–75. 10.1378/chest.11-2246 22281799

[221] Hu Q, Luo W, Huang L, Huang R, Chen R, Gao Y. Multiorgan protection of remote ischemic perconditioning in valve replacement surgery. J Surg Res. 2016; 200:13–20. 10.1016/j.jss.2015.06.053 26205311

[222] Li C, Li YS, Xu M, Wen SH, Yao X, Wu Y, Huang CY, Huang WQ, Liu KX. Limb remote ischemic preconditioning for intestinal and pulmonary protection during elective open infrarenal abdominal aortic aneurysm repair: a randomized controlled trial. Anesthesiology. 2013; 118:842–52. 10.1097/ALN.0b013e3182850da5 23353795

[223] Li C, Xu M, Wu Y, Li YS, Huang WQ, Liu KX. Limb remote ischemic preconditioning attenuates lung injury after pulmonary resection under propofol-remifentanil anesthesia: a randomized controlled study. Anesthesiology. 2014; 121:249–59. 10.1097/ALN.0000000000000266 24743579

[224] García-de-la-Asunción J, Bruno L, Perez-Griera J, Galan G, Morcillo A, Wins R, García-Del-Olmo E, Guijarro R, Sarriá B, Martí F, Soro M, Belda FJ. Remote ischemic preconditioning decreases oxidative lung damage after pulmonary lobectomy: a single-center randomized, double-blind, controlled trial. Anesth Analg. 2017; 125:499–506. 10.1213/ANE.0000000000002065 28504995

[225] Lin E, Snell GI, Levvey BJ, Mifsud N, Paul M, Buckland MR, Gooi J, Marasco S, Sharland AF, Myles PS. Safety, feasibility, and effect of remote ischemic conditioning in patients undergoing lung transplantation. J Heart Lung Transplant. 2014; 33:1139–48. 10.1016/j.healun.2014.04.022 25016922

[226] Kanoria S, Robertson FP, Mehta NN, Fusai G, Sharma D, Davidson BR. Effect of remote ischaemic preconditioning on liver injury in patients undergoing major hepatectomy for colorectal liver metastasis: a pilot randomised controlled feasibility trial. World J Surg. 2017; 41:1322–30. 10.1007/s00268-016-3823-4 27933431 PMC5394145

[227] Robertson FP, Goswami R, Wright GP, Fuller B, Davidson BR. Protocol for a prospective randomized controlled trial of recipient remote ischaemic preconditioning in orthotopic liver transplantation (RIPCOLT trial). Transplant Res. 2016; 5:4. 10.1186/s13737-016-0033-4 27054029 PMC4822296

[228] Robertson FP, Magill LJ, Wright GP, Fuller B, Davidson BR. A systematic review and meta-analysis of donor ischaemic preconditioning in liver transplantation. Transpl Int. 2016; 29:1147–54. 10.1111/tri.12849 27564598

[229] Kraemer R, Lorenzen J, Kabbani M, Herold C, Busche M, Vogt PM, Knobloch K. Acute effects of remote ischemic preconditioning on cutaneous microcirculation--a controlled prospective cohort study. BMC Surg. 2011; 11:32. 10.1186/1471-2482-11-32 22111972 PMC3231986

[230] Kolbenschlag J, Sogorski A, Harati K, Daigeler A, Wiebalck A, Lehnhardt M, Kapalschinski N, Goertz O. Upper extremity ischemia is superior to lower extremity ischemia for remote ischemic conditioning of antero-lateral thigh cutaneous blood flow. Microsurgery. 2015; 35:211–17. 10.1002/micr.22336 25278482

[231] Kolbenschlag J, Sogorski A, Timmermann C, Harati K, Daigeler A, Hirsch T, Goertz O, Lehnhardt M. Ten minutes of ischemia is superior to shorter intervals for the remote ischemic conditioning of human microcirculation. Clin Hemorheol Microcirc. 2017; 66:239–48. 10.3233/CH-170268 28482626

[232] Johnsen J, Pryds K, Salman R, Løfgren B, Kristiansen SB, Bøtker HE. The remote ischemic preconditioning algorithm: effect of number of cycles, cycle duration and effector organ mass on efficacy of protection. Basic Res Cardiol. 2016; 111:10. 10.1007/s00395-016-0529-6 26768477

[233] Berger MM, Köhne H, Hotz L, Hammer M, Schommer K, Bärtsch P, Mairbäurl H. Remote ischemic preconditioning delays the onset of acute mountain sickness in normobaric hypoxia. Physiol Rep. 2015; 3:e12325. 10.14814/phy2.12325 25742960 PMC4393159

[234] de Groot PC, Thijssen DH, Sanchez M, Ellenkamp R, Hopman MT. Ischemic preconditioning improves maximal performance in humans. Eur J Appl Physiol. 2010; 108:141–46. 10.1007/s00421-009-1195-2 19760432 PMC2793394

[235] Bailey TG, Birk GK, Cable NT, Atkinson G, Green DJ, Jones H, Thijssen DH. Remote ischemic preconditioning prevents reduction in brachial artery flow-mediated dilation after strenuous exercise. Am J Physiol Heart Circ Physiol. 2012; 303:H533–38. 10.1152/ajpheart.00272.2012 22730390

[236] Jean-St-Michel E, Manlhiot C, Li J, Tropak M, Michelsen MM, Schmidt MR, McCrindle BW, Wells GD, Redington AN. Remote preconditioning improves maximal performance in highly trained athletes. Med Sci Sports Exerc. 2011; 43:1280–86. 10.1249/MSS.0b013e318206845d 21131871

[237] Shaked G, Czeiger D, Abu Arar A, Katz T, Harman-Boehm I, Sebbag G. Intermittent cycles of remote ischemic preconditioning augment diabetic foot ulcer healing. Wound Repair Regen. 2015; 23:191–96. 10.1111/wrr.12269 26083360

[238] Liu X, Sha O, Cho EY. Remote ischemic postconditioning promotes the survival of retinal ganglion cells after optic nerve injury. J Mol Neurosci. 2013; 51:639–46. 10.1007/s12031-013-0036-2 23733254

[239] Zhang X, Jizhang Y, Xu X, Kwiecien TD, Li N, Zhang Y, Ji X, Ren C, Ding Y. Protective effects of remote ischemic conditioning against ischemia/reperfusion-induced retinal injury in rats. Vis Neurosci. 2014; 31:245–52. 10.1017/S0952523814000121 24735565

[240] Brandli A, Johnstone DM, Stone J. Remote ischemic preconditioning protects retinal photoreceptors: evidence from a rat model of light-induced photoreceptor degeneration. Invest Ophthalmol Vis Sci. 2016; 57:5302–13. 10.1167/iovs.16-19361 27727393

[241] Kottenberg E, Musiolik J, Thielmann M, Jakob H, Peters J, Heusch G. Interference of propofol with signal transducer and activator of transcription 5 activation and cardioprotection by remote ischemic preconditioning during coronary artery bypass grafting. J Thorac Cardiovasc Surg. 2014; 147:376–82. 10.1016/j.jtcvs.2013.01.005 23465551

[242] Bautin A, Datsenko S, Tashkhanov D, Gordeev M, Rubinchik V, Kurapeev D, Galagudza M. ASSA13-08-15 influence of the anaesthesia technique on the cardioprotective effects of the remote ischemic preconditioning in the patients undergoing the aortic valve replacement. Heart. 2013 (Suppl 1); 99:A40.

[243] Roubille F, Lairez O, Mewton N, Rioufol G, Ranc S, Sanchez I, Cung TT, Elbaz M, Piot C, Ovize M. Cardioprotection by clopidogrel in acute ST-elevated myocardial infarction patients: a retrospective analysis. Basic Res Cardiol. 2012; 107:275. 10.1007/s00395-012-0275-3 22718009

[244] Przyklenk K. Efficacy of cardioprotective ‘conditioning’ strategies in aging and diabetic cohorts: the co-morbidity conundrum. Drugs Aging. 2011; 28:331–43. 10.2165/11587190-000000000-00000 21542657

[245] Cohen MV, Downey JM. Combined cardioprotectant and antithrombotic actions of platelet P2Y12 receptor antagonists in acute coronary syndrome: just what the doctor ordered. J Cardiovasc Pharmacol Ther. 2014; 19:179–90. 10.1177/1074248413508465 24298192

[246] Sloth AD, Schmidt MR, Munk K, Schmidt M, Pedersen L, Sørensen HT, Bøtker HE, and CONDI Investigators. Impact of cardiovascular risk factors and medication use on the efficacy of remote ischaemic conditioning: post hoc subgroup analysis of a randomised controlled trial. BMJ Open. 2015; 5:e006923. 10.1136/bmjopen-2014-006923 25838505 PMC4390720

[247] Pryds K, Terkelsen CJ, Sloth AD, Munk K, Nielsen SS, Schmidt MR, Bøtker HE, and CONDI Investigators. Remote ischaemic conditioning and healthcare system delay in patients with ST-segment elevation myocardial infarction. Heart. 2016; 102:1023–28. 10.1136/heartjnl-2015-308980 26911520

[248] Pryds K, Bøttcher M, Sloth AD, Munk K, Rahbek Schmidt M, Bøtker HE, and CONDI Investigators. Influence of preinfarction angina and coronary collateral blood flow on the efficacy of remote ischaemic conditioning in patients with ST segment elevation myocardial infarction: post hoc subgroup analysis of a randomised controlled trial. BMJ Open. 2016; 6:e013314. 10.1136/bmjopen-2016-013314 27884851 PMC5168541

