# Supplementary material for: Remote ischemic conditioning: a promising therapeutic intervention for multi-organ protection
Source: Aging (Albany NY). 2018 Aug 16;10(8):1825–55. doi: 10.18632/aging.101527 (PMC6128414; doi:10.18632/aging.101527)
Supplement: Supplemental Figure 1 [file aging-10-101527-s001.docx]

**
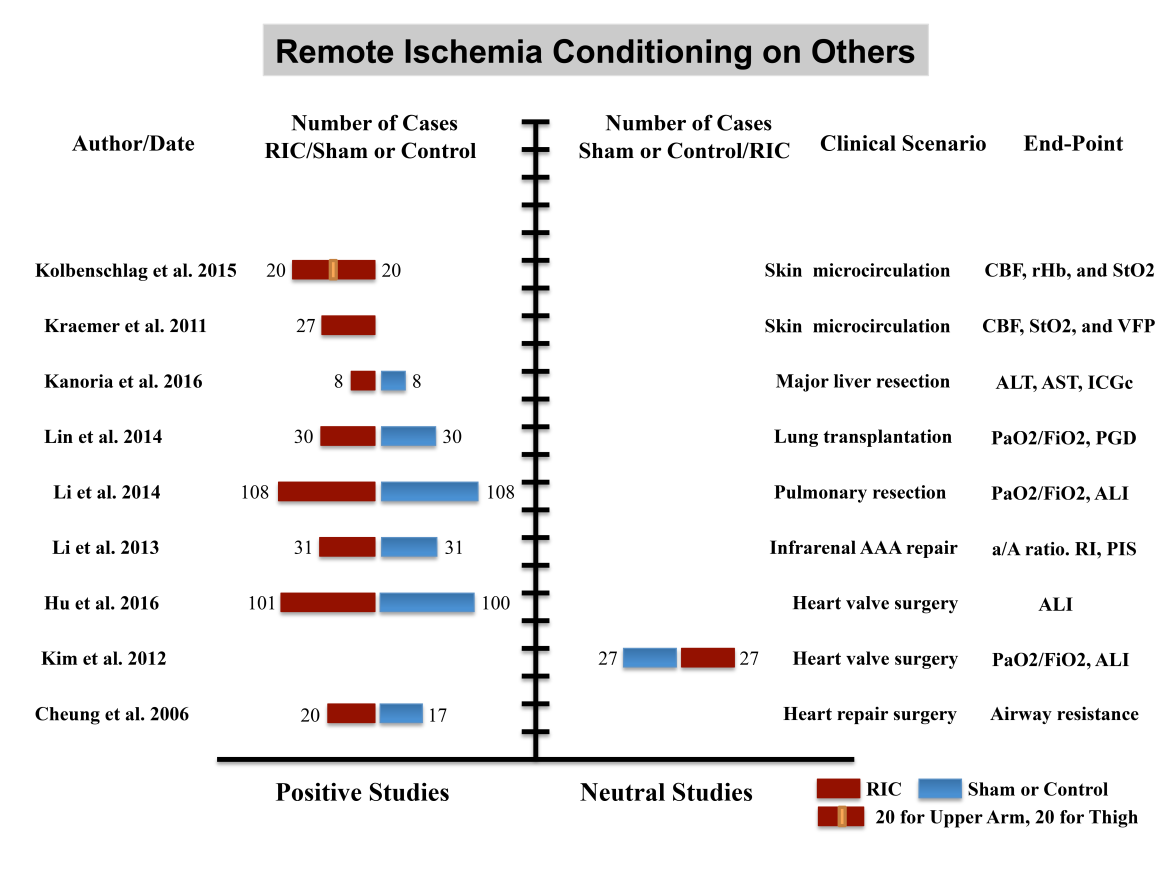
**

**Supplemental Figure 1. Clinical Trials of Remote Ischemic Conditioning (RIC) on other organs.**

Abbreviations: AAA, abdominal aortic aneurysm; rHb, relative hemoglobin content; StO2, oxygen saturation; VFP, venous filling pressure; ALT, alanine aminotransferase; AST, aspartate aminotransferase; ICGc, indocyanine green clearance; PaO2/FiO2, partial pressure of oxygen/fraction of inspired oxygen; PGD, primary graft dysfunction; ALI, acute lung injury; RI, respiratory index; PIS, pulmonary injury severity.
