# Supplementary material for: Remote ischemic conditioning: a promising therapeutic intervention for multi-organ protection
Source: Aging (Albany NY). 2018 Aug 16;10(8):1825–55. doi: 10.18632/aging.101527 (PMC6128414; doi:10.18632/aging.101527)
Supplement: Supplemental Table 1-4 [file aging-10-101527-s002.docx]

**Supplemental Table 1. Clinical Evidence of RIC in Brain Protection.**

| **Study** | **Clinical Scenario** | **Group Subject, Number (RIC/control)** | **RIC Regimen** | **Major Findings** |
| --- | --- | --- | --- | --- |
| Hougaard  et al. (2014) | AIS | Adult patients with symptoms of acute stroke, 171 (91/80) | -Upper arm  -4 cycles I/R (5/5 min) during transfer before receiving rtPA | -Reduction in the risk of tissue infarction after 1 m (based on voxelwise analysis after the adjustment of baseline severity of hypoperfusion)  -No obvious change in terms of penumbral salvage, infarct size and infarct progression (1 m) or clinical outcomes (3 m) |
| England  et al. (2017) | AIS | Adult patients with symptoms of acute stroke, 26 (13/13) | -Upper arm  -4 cycles I/R (5/5 min) on the stroke unit | -Suggest the safety and feasibility of RIC  -Significantly decreased NIHSS score (3 m)  -Significantly increased plasma HSP27 and pHSP27 (4 d)  -No obvious change in terms of plasma S100-β, MMP-9, and endocannabinoids |
| Koch  et al. (2011) | ASH | Patients with ASH due to ruptured cerebral aneurysms, 33 | -Upper arm or thigh  -3 cycles I/R (5/5 min) every 24 to 48 h from the time of enrollment to day 14 (or discharge if before day 14) | Suggest the safety and feasibility of RIC, even at ischemia times of 10 min |
| Bilgin-Freiert  et al. (2012) | ASH | Adult patients with ASH (ruptured aneurysm secured by clipping or coiling, all with external ventricular catheters placed), 7 (7/0) | -Thigh  -3-4 sessions of 4 cycles I/R (5/5 min) on non-consecutive days during day 4–12 after aneurysm rupture | -Significant increases in the lactate/pyruvate ratio and lactate immediately after the procedure  -No complications associated with the procedure during a follow-up of 29 days |
| Gonzalez  et al. (2013) | ASH | Adult patients with ASH (ruptured aneurysm secured by clipping or coiling, all with external ventricular catheters placed), 4 (4/0) | -Thigh  -3-4 sessions of 4 cycles I/R (5/5 min) on non-consecutive days during day 2–12 after aneurysm rupture | -Significant increase in the ICP and reduction in  MCA mean velocities immediately after the procedure  -Significant reductions in the lactate/pyruvate ratio and glycerol (persisted for 25-54 h) |
| Gonzalez  et al. (2014) | ASH | Adult patients with ASH (ruptured aneurysm secured by clipping or coiling, all with external ventricular catheters placed), 20 (20/0) | -Thigh  -4 cycles I/R (5/5 min) on non-consecutive days until 4 sessions completed or discharged or clinical endpoint completed | Suggest the safety and feasibility of RIC |
| Nikkola  et al. (2015) | ASH | Adult patients with ASH (ruptured aneurysm secured by clipping or coiling, all with external ventricular catheters placed), 37 (13/24) | -Thigh  -4 sessions of 4 cycles I/R (5/5 min) on non-consecutive days during day 2–12 after aneurysm rupture | -The first study to investigate genome-wide expression and methylation changes in SAH patients after RIC  -Obtain the evidence for coordinated expression and methylation changes of key genes in mitotic cell cycle, defense, and inflammatory responses |
| Laiwalla  et al. (2016) | ASH | Adult patients with ASH (ruptured aneurysm secured by clipping or coiling), 82 (21/61) | -Thigh  -4 sessions of 4 cycles I/R (5/5 min) on non-consecutive days during day 2-12 after aneurysm rupture | -Independently associated with good clinical outcomes  -Display a trend toward lower incidence of stroke and death |
| Meng  et al. (2012) | sIAS | Adult patients (younger than 80 years of age) with confirmed sIAS that presented within 30 days of an ischemic stroke or TIA, 68 (38/30) | -Bilateral upper arms  -5 cycles I/R (5/5 min) twice daily for consecutive 300 days | -18% and 19% reduction in the incidence of recurrent stroke at 90 and 300 days, respectively  -Shortened average time to recovery (depicted by mRS score 0-1)  -Significant improvement of cerebral perfusion status |
| Meng  et al. (2015) | sIAS | Elderly patients (above 80 years of age) with confirmed sIAS that presented within 7 days of an ischemic stroke or TIA, 58 (30/28) | -Bilateral upper arms  -5 cycles I/R (5/5 min) twice daily for consecutive 180 days | -No adverse effect on blood pressure, heart rate, local skin and muscle etc. (within 1 m)  -Ameliorations in plasma biomarkers of inflammation and coagulation such as reduction in hs-CRP, IL-6, PAI-1, leukocyte count, and PARs, as well as an elevation in TPA (within 1 m)  -Inhibition of stroke recurrence (within 6 m) |
| Wei  et al. (2016) | sIAS | Adult patients (18-45 years of age) with confirmed sIAS that present an ischemic stroke or TIA, 100 (50/50) | -Bilateral upper arms  -5 cycles I/R (5/5 min) twice daily for consecutive 180 days | -Ongoing clinical trial  -Primary end point: mean change in collateral circulation (within 6 m)  -Secondary end points: incidence of recurrent stroke or TIA, NIHSS and ADL scores, and serum VEGF and bFGF levels (within 6 m) |
| Hou  et al. (2016) | sIAS | Adult patients (40-80 years of age) with confirmed sIAS that present an ischemic stroke (within 30 days) or TIA (within 15 days), 3000 (1500/1500) | -Bilateral upper arms  -5 cycles I/R (5/5 min) once daily for consecutive 12 m | -Ongoing multicenter clinical trial  -Primary end point: first occurrence of ischemic stroke event during the study period  -Secondary end points: occurrence of composite fatal and nonfatal stroke, MI, TIA and death  -Tertiary end points: NIHSS, mRS, and BI scores after 12 m |
| Walsh  et al. (2010) | CEA | Adult patients undergoing CEA, 70 (34/36) | -Thigh  -2 cycles (one in each leg) I/R (10/10 min) on the day of surgery | -Display a trend toward fewer saccadic latency deteriorations  -No adverse events related to the procedure |
| Zhao  et al. (2017) | CAS | -Adult patients undergoing CAS, 189 (63/126)  -Three groups: group A (63 for RIC); group B (63 for sham RIC); group C (63 for control) | -Bilateral upper arms  -5 cycles I/R (5/5 min) twice daily for 2 weeks before CAS | -Significant reduced incidence of new DWI lesions (group A: 15.87% vs. group B: 36.51% vs. group C: 41.27%)  -Significant reductions in the volumes of lesions  -No obvious change in terms of plasma NSE, S-100B levels and hs-CRP (within 24 h)  -No adverse events related to the procedure |
| Joseph  et al. (2015) | TBI | Adult patients with blunt TBI that presented GCS score of 8 or lower, 40 (20/20) | -Upper arm  -4 cycles I/R (5/5 min) within 1 h of admission | -Significant reductions in the serum levels of NSE and S-100β (at 6 and 24 h)  -No obvious complications associated with the procedure |
| Mi  et al. (2016) | CSVD | Adult patients (40-80 years of age) diagnosed with CSVD, 17 (9/8) | -Bilateral upper arms  -5 cycles I/R (5/5 min) twice daily for consecutive 12 m | -Acceleration on MFV of the left MCA  -Reductions in DHI and WMLs |
| Wang  et al. (2017) | CSVD | Adult patients (45-80 years of age) diagnosed with CSVD, 30 (14/16) | -Bilateral upper arms  -5 cycles I/R (5/5 min) twice daily for consecutive 12 m | -Significant reductions in the WMLs  -Obvious improvements in the visuospatial and executive ability (12 m)  -Significant decreases in the levels of triglyceride, TC, LDL, homocysteine (12 m) |
| REM-PROTECT | CSVD | Adult patients (above 40 years of age) diagnosed with SVD, 60 | -Bilateral upper arms  -4 cycles I/R (5/5 min) once or twice daily for consecutive 12 m | -Ongoing clinical trial  -Primary end points: feasibility such as behavioral adherence, physiologic attainment of limb ischemia and patient subject complaints (12 m)  -Secondary end points: Safety (adverse events within 24 m); progression of WMLs (12 m) |
| **Abbreviation:** AIS, acute ischemic stroke; HSP, heat shock protein; pHSP, phosphorylated heat shock protein; MMP-9, matrix metalloproteinase-9; ASH, acute subarachnoid hemorrhage; sIAS, symptomatic intracranial arterial stenosis; TIA, transient ischemic attack; hs-CRP, high sensitive-C reactive protein; IL-6, interleukin-6; PAI-1, plasminogen activator inhibitor-1; PAR, platelet aggregation rate; TPA, tissue plasminogen activator; NIHSS, National Institutes of Health Stroke Scale; ADL, activities of daily living; VEGF, vascular endothelial growth factor; bFGF, basic fibroblast growth factor; MI, myocardial infarction; mRS, modified Rankin scale; BI, Barthel index; CEA, carotid endarterectomy; CAS, carotid artery stenting; DWI, diffusion-weighted imaging; NSE, neuron-specific enolase; TBI, traumatic brain injury; GCS, Glasgow Coma Scale; CSVD, cerebral small vessel disease; MFV, mean flow velocity; MCA, middle cerebral artery; DHI, dizziness handicap inventory; WMLs, white matter lesions; TC, total cholesterol; total low-density lipoprotein, LDL | | | | |

**Supplemental Table 2. Clinical Evidence of RIC in Heart Protection.**

| **Study** | **Clinical Scenario** | **Group Subject, Number (RIC/control)** | **RIC Regimen** | **Major Findings** |
| --- | --- | --- | --- | --- |
| Hausenloy  et al. (2007) | -CABG  -Blood cardioplegia or ICCF | Adult patients with CAD,  57 (27/30) | -Upper arm  -3 cycles I/R (5/5 min) after anesthesia but before surgery | 43% reduction in cTnT (72 h AUC) |
| Venugopal  et al. (2009) | -CABG with or without aortic valve surgery  -Blood cardioplegia | Adult patients with CAD,  45 (23/22) | -Upper arm  -3 cycles I/R (5/5 min) after anesthesia but before surgery | 42% reduction in cTnT (72 h AUC) |
| Thielmann  et al. (2010) | -CABG  -Crystalloid cardioplegia | Adult patients with CAD (no diabetic patients),  53 (27/26) | -Upper arm  -3 cycles I/R (5/5 min) after anesthesia but before surgery | 45% reduction in cTnI (72 h AUC) |
| Wagner  et al. (2010) | -CABG with or without aortic valve surgery  -Crystalloid cardioplegia | Adult patients with CAD,  66 (32/34) | -Upper arm  -3 cycles I/R (5/5 min) after anesthesia but before surgery | -12% reduction in cTnI (8 h troponin I peak)  -Effect of RIC abolished by tramadol |
| Ali  et al. (2010) | -CABG | Adult patients with CAD,  100 (50/50) | -Upper arm  -3 cycles I/R (5/5 min) after anesthesia but before surgery | Reductions in CK-MB levels (8, 16, 24 and 48 hours after surgery) |
| Thielmann  et al. (2013) | -CABG  -Crystalloid cardioplegia | Adult patients with CAD,  329 (162/167) | -Upper arm  -3 cycles I/R (5/5 min) after anesthesia but before surgery | -17% reduction in cTnI (72 h AUC)  -78% reduction in all-cause mortality |
| Candilio  et al. (2015) | -CABG  -Crystalloid cardioplegia | Adult patients with CAD,  180 (90/90) | -Upper arm and thigh  -2 cycles I/R (5/5 min) after anesthesia but before surgery | -26% reduction in hsTnT (72 h)  -54% reduction in postoperative atrial fibrillation  -Shorten ICU stay by 1 day |
| Gunaydin  et al. (2000) | -CABG | Adult male patients with CAD,  8(4/4) | -Upper arm  -2 cycles I/R (3/2 min) | No obvious change in cardiac enzymes (collected via coronary perfusion catheter 5 min after declamping) |
| Rahman  et al. (2010) | -CABG  -Blood cardioplegia | Adult patients with CAD,  162 (80/82) | -Upper arm  -3 cycles I/R (5/5 min) after anesthesia and following surgery incision | No obvious change in cTnT (48 h AUC), dialysis need, arrhythmias, inotrope support etc. |
| Lucchinetti  et al. (2012) | -CABG  -Blood cardioplegia | Adult patients with CAD,  162 (27/28) | -Thigh  -4 cycles I/R (5/5 min) after anesthesia but before surgery | -No obvious change in hsTnT, N-terminal pro-brain natriuretic peptide, hs-CRP, and S-100 etc.  -Suggest that RIC ineffective in presence of isoflurane |
| Young  et al. (2012) | -CABG with or without aortic valve surgery | High risk adult patients with CAD,  96 (48/48) | -Upper arm  -3 cycles I/R (5/5 min) after anesthesia but before surgery | No obvious change in hsTnT (6 h and 12 h) |
| Hausenloy  et al. (2015) | -CABG with or without aortic valve surgery  -Blood cardioplegia | High risk adult patients with CAD,  1612 (801/811) | -Upper arm  -4 cycles I/R (5/5 min) after anesthesia but before surgery | -No obvious change in the primary end points such as death, MI, coronary revascualarisation and stroke within 12 m  -No obvious change in secondary end points such as hsTnT (72 h), inotrope score, kidney injury, ICU stay and quality of life etc.  -Largest multicenter clinical trial revealing ineffectiveness of RIC on 1 year outcome |
| Meybohm  et al. (2015) | -CABG with or without valve surgery  -Blood cardioplegia | Adult patients with CAD,  1385 (692/693) | -Upper arm  -4 cycles I/R (5/5 min) after anesthesia but following surgery incision | -No obvious change in the primary end points including death, MI, stroke and renal failure up to the time of hospital discharge  -No obvious change in secondary end points such as troponin release, ICU stay, atrial fibrillation, and post-operative delirium |
| Hoole  et al. (2009) | Selective PCI | Adult patients with CAD (stable),  202 (104/98) | -Upper arm  -3 cycles I/R (5/5 min) immediately before PCI | -Attenuation in the procedure-related cTnI (24 h) release  -A reduction in MACCE rate at 6 m |
| Ahmed  et al. (2013) | Selective PCI | Adult patients with CAD (stable),  149 (77/72) | -Upper arm  -3 cycles I/R (5/5 min) immediately before PCI | Attenuation in the procedure-related cTnT (16 h) release |
| Luo  et al. (2013) | Selective PCI | Adult patients with CAD (stable),  205 (101/104) | -Upper arm  -3 cycles I/R (5/5 min) immediately before PCI | -48% reduction in hs-cTnI (16 h) release  -15% reduction in the incidence of post-procedure (type 4a) MI |
| Davies  et al. (2013) | Selective PCI | Adult patients with CAD (stable),  192 (95/97) | -Upper arm  -3 cycles I/R (5/5 min) immediately before PCI | 13% reduction in MACCE at 6 years |
| Zografos  et al. (2014) | Selective PCI | Adult patients with CAD (stable),  94 (47/47) | -Upper arm  -1 cycle I/R (5/5 min) immediately before PCI | -80% reduction in cTnI (24 h) release  -56% reduction in the incidence of PCI-related MI |
| Liu  et al. (2014) | Selective PCI | Adult patients with CAD (stable),  200 (98/102) | -Upper arm  -3 cycles I/R (5/5 min) 18 h before PCI | Significant reduction in CK, CK-MB, cTnI (24 h) release and rate of adverse events at 6 m |
| Iliodromitis  et al. (2006) | Selective PCI | Adult patients with CAD (stable),  41 (20/21) | -Bilateral upper arms  -3 cycles I/R (5/5 min) immediately before PCI | Increase in CK-MB and cTnI (24 h and 48 h) |
| Prasad  et al. (2013) | Selective PCI | Adult patients with CAD (75% stable vs. 25% unstable),  95 (47/48) | -Upper arm  -3 cycles I/R (3/3 min) immediately before PCI | -No obvious change in the frequency of post-PCI myonecrosis (defined as a peak postprocedural cTnT ≥0.03 ng/dL)  -No obvious change in post-hsCRP and circulating EPC |
| Xu  et al. (2014) | Selective PCI | Diabetic patients aged above 65 years of age with CAD (75% stable vs. 25% unstable),  200 (102/98) | -Upper arm  -3 cycles I/R (5/5 min) immediately before PCI | No obvious change in hscTnI (16 h) and the incidence of post-procedure (type 4a) MI (Despite decline trend) |
| Lavi  et al. (2014) | Selective PCI | Adult patients with CAD (72% stable vs. 28% unstable),  360 (240/120) | -Upper arm or thigh  -3 cycles I/R (5/5 min) immediately after PCI | -No obvious change in hscTnT (6 or 18-24 h)  -No difference between upper and low limb RIC |
| Moretti  et al. (2015) | Selective PCI | Adult patients with CAD (stable),  555 | -Upper arm  -3 cycles I/R (5/5 min) immediately before PCI | -Ongoing multicenter clinical trial  -Primary end point: incidence of CIN  -Secondary end point: periprocedural myocardial injury |
| Rentoukas  et al. (2010) | PPCI | -Adult patients with STEMI,  93 (66/30)  -Three groups: group A (33 for RIC); group B (33 for RIC plus morphine); group C (30 for control) | -Upper arm  -3 cycles I/R (4/4 min) at the hospital before PPCI | -Higher proportion of patients in group A and group B than group C achieved full ST-segment resolution  -Lower peak TnI in group B and group C  -Additive effect by morphine |
| Botker  et al. (2010) | PPCI | Adult patients with STEMI,  142 (73/69) | -Upper arm  -4 cycles I/R (5/5 min) during transfer to the hospital before PPCI | 20% increase in myocardial salvage index (at 1 m) |
| Munk  et al. (2010) | PPCI | Adult patients with STEMI,  218 (108/110) | -Upper arm  -4 cycles I/R (5/5 min) during transfer to PPCI | Significant preservation of LV function among patients with anterior infarcts or extensive AAR ≥35% of LV (day 1 and 30) |
| Crimi  et al. (2013) | PPCI | Adult patients with STEMI,  100 (50/50) | -Thigh  -3 cycles I/R (5/5 min) at the beginning of reperfusion at the moment of PPCI | -20% reduction in CK-MB (72 h AUC)  -Significant improvement of T2-weighted edema volume and ST-segment resolution >50% |
| Sloth  et al. (2014) | PPCI | Adult patients with STEMI,  251 (166/167) | -Upper arm  -4 cycles I/R (5/5 min) during transfer to the hospital before PPCI | -12% reduction in MACCE at 3.8 year  -First clinical trial to assess the effect of RIC on long-term end points after PPCI |
| White  et al. (2015) | PPCI | Adult patients with STEMI,  83 (43/40) | -Upper arm  -4 cycles I/R (5/5 min) at hospital before PPCI | -27% and 19% reduction in MI size and myocardial edema, respectively  -Significantly reduced hscTnT and improved myocardial salvage |
| Eitel  et al. (2015) | PPCI | -Adult patients with STEMI,  696  -Three groups: group A (232 for RIC plus IpostC); group B (232 for IpostC); group C (232 for control) | -Upper arm  -4 cycles I/R (5/5 min) at hospital before PPCI plus IpostC | -Improved myocardial salvage (in group A rather than group B and C)  -No obvious change in MVO and clinical outcomes such as death, heart failure, and reinfarction at 6 m |
| Aspar  et al. (2018) | PPCI | -Adult patients with STEMI,  448 (231/217) | -Thigh  -3 cycles I/R (5/5 min) at hospital before PPCI | Significant reduction in cardiac mortality and HHF (approximately 2 years) |
| Hausenloy  et al. (2015) | PPCI | Adult patients with STEMI,  4300 | -Upper arm  -4 cycles I/R (5/5 min) at hospital before PPCI | -Ongoing multicenter clinical trial  -Primary end points: cardiac death and HHF at 1 year |
| **Abbreviation:** CABG, coronary artery bypass graft surgery; ICCF, intermittent cross-clamp fibrillation; CAD, coronary artery disease; Tn, troponin; AUC, area under the curve; CK-MB, creatine kinase-myocardial band; ICU, intensive care unit; hs-CRP, high sensitive-C reactive protein; MI, myocardial infarction; PCI, percutaneous coronary intervention; EPC, endothelial progenitor cells; MACCE, major adverse cardiac and cerebral event; CK, creatine kinase; CIN, contrast-induced nephropathy; PPCI, primary percutaneous coronary intervention; STEMI, ST-segment elevation myocardial infarction; LV, left ventricular; AAR, myocardial area at risk; IpostC, ischemic postconditioning; MVO, microvascular obstruction; HHF, hospitalization for heart failure | | | | |

**Supplemental Table 3. Clinical Evidence of RIC in Kidney Protection.**

| **Study** | **Clinical Scenario** | **Group Subject, Number (RIC/control)** | **RIC Regimen** | **Major Findings** |
| --- | --- | --- | --- | --- |
| Venugopal  et al. (2010) | CABG with or without valve surgery | Non-diabetic patients with CAD, 78 (38/40) | -Upper arm  -3 cycles I/R (5/5 min) after anesthesia but before surgery | Reduction in the incidence of ARD (within 72 h) |
| Er  et al. (2012) | Selective PCI | -Adult patients with CAD, 100 (50/50)  -High risk of developing CIN based on Mehran risk score | -Upper arm  -4 cycles I/R (5/5 min) immediately before PCI | -28% reduction in the incidence of CIN (within 48 h)  -Lower levels of serum creatinine, cystatin C and urinary NGAL (within 48 h)  -Reduction in the composite cardiovascular end points (within 6 w) |
| Igarashi  et al. (2013) | Selective PCI | Adult patients with low to moderate CKD, 60 (30/30) | -Upper arm  -4 cycles I/R (5/5 min) immediately before PCI | -19% reduction in the L-FABP based CIN  -No difference in inflammatory biomarkers |
| Menting  et al. (2015) | Contrast induction | -Adult patients at risk of CIN, 72 (36/36)  -Standard hydration plus RIC vs. standard hydration plus sham | -Upper arm  -4 cycles I/R (5/5 min) immediately before PCI | Significant reduced level of serum creatinine (up to 72 h) at high risk of CIN (Mehran score ≥ 11) |
| Balbir  et al. (2016) | Selective PCI | Diabetic patients with preexisting CIN and chest pain, 102 (51/51) | -Upper arm  -3 cycles I/R (5/5 min) 30 min before PCI | No obvious change with regard to the incidence of CIN, and serial changes in creatinine, NGAL, cTnT, CK-MB and hs-CRP |
| Zimmerman  et al. (2011) | CABG with or without valve surgery | Adult patients with CAD, 118 (59/59) | -Thigh  -3 cycles I/R (5/5 min) after anesthesia but before surgery | 27% reduction in the incidence of ARD (within 48 h) |
| Gallagher  et al. (2015) | CABG with or without valve surgery | Adult patients with CAD and CKD, 86 (43/43) | -Upper arm  -3 cycles I/R (5/5 min) after anesthesia but before surgery | No obvious change with regard to the incidence of ARD (within 48 h), as well as serum or urinary biomarkers of renal or cardiac injury |
| Zarbock  et al. (2015) | Cardiac surgery  (comorbidities and complex surgery procedures) | Adult patients at high risk of ARD, 240 (120/120) | -Upper arm  -3 cycles I/R (5/5 min) after anesthesia but before surgery | -15% reduction in the incidence of ARD (within 72 h)  -Less use of RRT and shortened ICU stay  -Reductions in the ARD biomarkers including urinary IGFBP-7A and TIM-2 |
| Chen  et al. (2013) | Elective living-donor renal transplantation | -Patients referred for renal transplantation, 60 (40/20)  -Three groups: group A (20 for RIC on the donor); group B (20 for RIC on the recipient); group C (20 for control) | -Thigh  -3 cycles I/R (5/5 min) | No obvious change with regard to the renal function and ARD biomarkers (within 72 h) |
| Nicholson  et al. (2015) | Elective living-donor renal transplantation | Patients referred for renal transplantation, 80 (40/40) | -Thigh  -4 cycles I/R (5/5 min) | No obvious improvement in the renal function measured by eGFR |
| MacAllister  et al. (2015) | Elective living-donor renal transplantation | -Patients referred for renal transplantation, 406 (307/99)  -Four groups: group A (99 for control); group B (102 for early RIC, immediately before surgery); group C (103 for late RIC, 24 h before surgery); group D (102 for dual RIC, both early and late) | -Upper arm  -4 cycles I/R (5/5 min)  -RIC performed on both the donor and recipient at two time points before transplantation | -Display a trend of increase in iohexol GFR (at 1 year) and stronger beneficial evidence of eGFR (at 3 m and 1 year) in group B  -No (additional) protective effects observed in group C and group D  -Safe and well tolerated |
| Wu  et al. (2014) | DCD renal transplantation | Patients referred for renal transplantation, 48 (24/24) | -Thigh (clamping the exposed external iliac artery)  -3 cycles I/R (5/5 min)  -RIC performed on the recipient before transplantation | -Significant reductions in the serum creatinine level (12 h, days 1-14) and urine NGAL (12 and 24 h)  -Increase in the eGFR (12 h, days 1-14) |
| Krogstrup  et al. (2016) | Deceased donor renal transplantation | -Patients with end renal disease referred for renal transplantation, 222 (109/113)  -Donor sources from both DCD and DBD | -Thigh  -4 cycles I/R (5/5 min)  -RIC performed on the recipient before transplantation | -Multicenter clinical trial  -No obvious difference with regard to tCr50 (30 days posttransplant) and the number of patients receiving dialysis (7 days posttransplant) |
| van den Akker et al. (2014) | DCD renal transplantation | -Patients with end renal disease referred for renal transplantation, 20  -20 patients for IpostC; 40 historical data as control; 11 contralateral kidneys as control | -Thigh (clamping the exposed external iliac artery)  -2 cycles I/R (1/1 min)  -IpostC performed on the recipient before transplantation | -First study of applying IpostC in human renal transplantation  -No obvious difference with regard to DGF and serum creatinine (3 m) |
| Park  et al. (2014) | HD | Chronic HD patients, 34 (17, 17) | -Upper arm  -3 cycles I/R (5/5 min) performed before each HD session (12 times) | -Display a trend of decrease in cTnT level from day 2, significant difference at 28 d |
| **Abbreviation:** CABG, coronary artery bypass graft surgery; CAD, coronary artery disease; ARD, acute renal dysfunction; PCI, percutaneous coronary intervention; CIN, contrast-induced nephropathy; NGAL, neutrophil gelatinase-associated lipocalin; CKD, chronic kidney disease; L-FABP, liver-type fatty acid-binding protein; Tn, troponin; CK-MB, creatine kinase-myocardial band; hs-CRP, high sensitive-C reactive protein; RRT, renal replacement therapy; ICU, intensive care unit; IGFBP-7, insulinlike growth factor-binding protein-7; TIM-2: tissue inhibitor of metalloproteinases-2; eGFR, estimated glomerular filtration rate; DCD, donation after cardiac death; DBD, donation after brain death; tCr50, the estimated time to a 50% decrease in baseline plasma creatinine; IpostC, ischemic postconditioning; DGF, delayed graft function; HD, hemodialysis | | | | |

**Supplemental Table 4. Clinical Evidence of RIC in other Organ Protection.**

| **Study** | **Clinical Scenario** | **Group Subject, Number (RIC/control)** | **RIC Regimen** | **Major Findings** |
| --- | --- | --- | --- | --- |
| Cheung  et al. (2006) | Heart repair surgery | Children patients with congenital heart defects, 37 (20/17) | -Upper arm  -4 cycles I/R (5/5 min) before surgery | -Lower airway resistance (at 6 h)  -Significant reduction in TNF-α (at 6 h) and increase in IL-10 (at 3 h) |
| Hu  et al. (2016) | Heart valve surgery | Patients with rheumatic heart disease, 201 (101/100) | -Thigh  -3 cycles I/R (5/5 min) during surgery | 14% reduction in the incidence of acute lung injury |
| Kim  et al. (2012) | Heart valve surgery | Patients with valvular heart disease, 54 (27/27) | -Thigh  -3 cycles I/R (10/10 min) after anesthesia and weaning from cardiopulmonary bypass | No obvious difference with regard to PaO2/ FiO2, incidence of acute lung injury and several inflammatory markers |
| Li  et al. (2013) | Elective open infrarenal AAA repair | Patients scheduled for open infrarenal AAA repair, 62 (31/31) | -Upper arm  -3 cycles I/R (5/5 min) before surgery | -Significant elevation in a/A ratio (at 8, 12 and 24 h) and reductions in RI as well as pulmonary injury severity (at 8 h) |
| Li  et al. (2014) | Elective thoracic pulmonary resection | Patients with lung cancer, 216 (108/108) | -Upper arm  -3 cycles I/R (5/5 min) before surgery | -Significant elevation in PaO2/FiO2 and reductions in serum levels of IL-6, TNF-α and malondialdehyde  -Reduced incidence of acute lung injury |
| Lin  et al. (2014) | Bilateral lung transplantation | Patients with lung cancer, 60 (30/30) | -Thigh  -3 cycles I/R (5/5 min) before allograft reperfusion | -A trend towards higher PaO2/FiO2 and lower PGD severity score  -Reduced incidence of rejection at both 3 m and 1 y follow-up |
| Kanoria  et al. (2016) | Major liver resection | Patients with colorectal liver metastasis, 16 (8/8) | -Thigh  -2 cycles I/R (10/10 min) before surgery | -Significant reduction in ALT and AST levels (immediately post-resection and at 24 h)  -Significantly reduced ICG clearance (immediately post-resection) |
| Robertson  et al. (2016) | Liver transplantation | Patients scheduled for elective deceased donor liver transplantation, 50 (25/25) | -Thigh  -3 cycles I/R (5/5 min) before surgery | -Ongoing clinical trial  -Primary end points: feasibility and safety  -Secondary end points such as reductions in AST levels, incidence of ARD or rejection, and circulating cytokine levels |
| Kraemer  et al. (2011) | Cutaneous microcirculation evaluation | Healthy subjects (25 males vs. 2 females, 18-35 years of age), 27 | -Upper arm  -3 cycles I/R (5/5 min) | -29% increase in tissue oxygen saturation and 35% increase in capillary blood flow (the third reperfusion phase)  -16% reduction in postcapillary VFP (the second reperfusion phase) |
| Kolbenschlag  et al. (2015) | Cutaneous microcirculation evaluation | -Healthy subjects (19-40 years of age), 40  -20 for upper arm, 20 for thigh | -Upper arm or thigh  -3 cycles I/R (5/10 min) | -Significant increases in cutaneous blood flow, rHb, and StO2 (both groups)  -Better cutaneous blood flow in the upper arm group |
| **Abbreviations:** TNF-α, tumor necrosis factor-α; PaO2/FiO2, partial pressure of oxygen/fraction of inspired oxygen; AAA, abdominal aortic aneurysm; RI, respiratory index; IL-6, interleukin-6; PGD, primary graft dysfunction; ALT, alanine aminotransferase; AST, aspartate aminotransferase; ICG, indocyanine green; ARD, acute renal dysfunction; VFP, venous filling pressure; rHb, relative hemoglobin content; StO2, oxygen saturation | | | | |
